# Evaluations of postoperative transitions in care for older adults: a scoping review

**DOI:** 10.1186/s12877-022-02989-6

**Published:** 2022-04-15

**Authors:** Emily Hladkowicz, Flavia Dumitrascu, Mohammad Auais, Andrew Beck, Sascha Davis, Daniel I. McIsaac, Jordan Miller

**Affiliations:** 1 School of Rehabilitation Therapy, Queen’s University, Kingston, Canada; 2 Department of Anesthesiology and Pain Medicine, The Ottawa Hospital, Room B311, 1053 Carling Ave, Mail Stop 249, Ottawa, ON K1Y 4E9 Canada; 3 Clinical Epidemiology Program, Ottawa Hospital Research Institute, Ottawa, Canada; 4 School of Medicine, Royal College of Surgeons in Ireland, Dublin, Ireland; 5 School of Epidemiology & Public Health, University of Ottawa, Ottawa, Canada; 6 Learning Services, The Ottawa Hospital, Ottawa, Canada; 7 Department of Anesthesiology and Pain Medicine, University of Ottawa, Ottawa, Canada

**Keywords:** Transitions in care, Surgery, Scoping review, Aging

## Abstract

**Background:**

Most people having major surgery are over the age of 65. The transition out of hospital is a vulnerable time for older adults, particularly after major surgery. Research on postoperative transitions in care is growing, but it is not clear how postoperative transitions are being evaluated. The objective of this scoping review was to synthesize processes and outcomes used to evaluate postoperative transitions in care for older adults.

**Methods:**

We conducted a scoping review that included articles evaluating a postoperative transition in care among adults aged > 65 having major elective surgery. We searched Medline (Ovid), EMBASE (Ovid), CINHAL, and Cochrane Central Register of Controlled Trials (CENTRAL) from their respective inception dates to April 6, 2021. We also searched The World Health Organization International Clinical Trials Registry Platform and ClinicalTrials.gov from their respective inception dates to April 6, 2021. Screening and data extraction was completed by reviewers in duplicate. Data relevant to study design and objective, intervention description, and process or outcome evaluations were extracted. Process evaluations were categorized using the Ideal Transitions in Care Framework, and outcome evaluations were categorized using the Institute for Healthcare Improvement Triple Aim Framework.

**Results:**

After screening titles and abstracts and full-text article review, we included 20 articles in our final synthesis. There was variability in the processes and outcomes used to evaluate postoperative transitions in care. The most common outcomes evaluated were health service utilization (*n* = 9), including readmission and Emergency Department visits, experiential outcomes (*n* = 9) and quality of life (*n* = 7). Process evaluations included evaluating the education provided to patients to promote self-management (*n* = 6), coordination of care among team members (*n* = 3) and outpatient follow-up (*n* = 3). Only two articles measured frailty, one article used theory to guide their evaluations and no articles engaged knowledge users.

**Conclusions:**

There is inconsistency in how postoperative transitions in care were evaluated. There is a need to use theories and to engage key stakeholders involved in postoperative transitions in care, including older adults and their caregivers, to identify the most appropriate approaches for developing and evaluating interventions to meaningfully improve care.

**Supplementary Information:**

The online version contains supplementary material available at 10.1186/s12877-022-02989-6.

## Introduction

The American Geriatrics Society defines transitions in care as “a set of actions designed to ensure the coordination and continuity of health care as patients transfer between different locations or different levels of care within the same location” [1]. The transition from one care setting to another can be challenging for patients and the healthcare system. Transitions can jeopardize patient safety [2] and can lead to unmet needs, low satisfaction with care and increased healthcare utilization [3]. In particular, the transition from hospital to home or a new location after surgery has been identified as a period of increased risk, especially for older adults who often have complex needs [4, 5]. This transition in care requires attention as most people having major surgery are over the age of 65 [6] and often have frailty, which places them at increased risk of poor postoperative outcomes [7, 8].

After major surgery, nearly one in seven older patients are readmitted to a hospital within 30 days of discharge [9]. Other adverse outcomes include discharge to long-term care, caregiver burden, and increased health care costs [10–13]. Even after minor surgery, older patients and their caregivers report feeling unprepared for managing the physical and emotional challenges in the postoperative period [12]. Because older people are often vulnerable, there may be unique elements requiring consideration during their postoperative transitions in care [14]. Accordingly, as the population ages, improving the quality of transitions out of hospital has been identified as an urgent priority in healthcare [15]. Therefore, transitional care interventions are increasingly being evaluated. A recent scoping review [16] examined hospital-to-home transitional care interventions for older adults leaving hospital and found that the most common outcomes evaluated were readmission and mortality. Of the 44 articles analyzed, none of the articles addressed transitions in older surgical patients, meaning that an important evidence gap exists in our understanding of what processes and outcomes are used to evaluate transitional care interventions for older adults after surgery. Importantly, once the process and outcome evaluations used in the literature are identified, we can determine how closely these evaluations align with the needs, priorities and preferences of older adults and their caregivers.

Given the unique and complex transitional care needs of older people having surgery and their caregivers, and their vulnerability during postoperative transitions, these findings will determine what processes and outcomes have been used to evaluate postoperative transitions in care and identify potential gaps in research for this population. The findings of this research will help inform evaluations in practice and future research on transitions in care for older adults having surgery. Therefore, our objective was to use scoping review methodology to synthesize the processes and outcomes used to evaluate postoperative transitions in care for older adults.

## Methods

We followed our registered protocol available on Open Science Framework (10.17605/OSF.IO/SAJRT). Consistent with a scoping review methodology, we used an iterative approach and documented all revisions and deviations from our original protocol in our protocol registration. We adhered to the PRISMA Extension for Scoping Reviews (PRISMA-Scr) guidelines (Additional File 1, [17] and followed the methodological process developed by Arskey and O’Malley and expanded by Levac [18, 19].

### Search strategy

A comprehensive search strategy was developed with a research librarian [SD] and peer-reviewed using the Peer Review of Electronic Search Strategies (PRESS) process (Additional File 2) [20]. We applied the search to the following databases: Medline (Ovid), EMBASE (Ovid), CINHAL, and Cochrane Central Register of Controlled Trials (CENTRAL). The searches in the electronic databases were carried out from their respective inception dates to April 6, 2021. We further searched reference lists of related systematic and scoping reviews, as well as included articles to identify relevant studies that could have been missed by our search. The World Health Organization International Clinical Trials Registry Platform and ClinicalTrials.gov were searched from their respective inception dates to April 6, 2021. Conference abstracts were eligible for inclusion if they met inclusion criteria. Articles were limited to those in English or French due to the linguistic abilities of the team.

When the full text of articles could not be found through multiple online databases or interlibrary loans by the research librarian, authors were emailed once and then a second time if they did not respond within 1 week. If the author did not respond to either request, the article was excluded.

### Inclusion criteria

Articles were included if: 1) the majority of the study participants were > 65 years of age (i.e., the mean age was > 65 years or > 50% of participants were > 65 years of age); 2) > 50% of study participants underwent elective inpatient surgery; 3) study participants experienced a transition from hospital to home or a new location after surgery; and 4) the study aimed to evaluate the process or outcome of the transition out of hospital after surgery. As care pathways differ between elective and urgent surgeries, this review focused on elective surgeries in order to ensure a clear definition of the target population. Postoperative transitional interventions could include interventions that began before surgery when the objective was to improve the postoperative transition in care. Any experimental or observational design (e.g., randomized controlled trials, prospective or retrospective cohort, case-control) with appropriate exposure and outcome data were included. Relevant qualitative articles evaluating the processes or outcomes of transitions in care were also included.

To maintain the integrity of the research question and to provide standardization for our inclusion criteria, articles were included when the authors either explicitly stated that their objective was to evaluate the processes or outcomes of a postoperative transition (going from hospital to home or a new location after surgery). As other terms are often used synonymously with transitions, articles that sought to evaluate processes or outcomes of a postoperative transition in care that used common synonyms for transitions were included. These terms included: integrated care, coordinated care, continuity of care and transitional care [21].

### Title and abstract screening

All articles were imported into DistillerSR software (Evidence Partners, Ottawa, Canada). The first 100 titles and abstracts were screened in duplicate by two reviewers (87% agreement was achieved). Remaining titles and abstracts were screened using a liberal accelerated approach [22]. To ensure that all articles were screened in duplicate prior to exclusion, all titles and abstracts that were identified as meeting exclusion criteria were reviewed by a second reviewer [EH, FD].

### Full text screening

All articles that were not excluded by both reviewers were advanced to full text review. Full text articles were reviewed in duplicate [EH, FD]. Disagreements were resolved through consensus, and where consensus could not be reached, a third reviewer was consulted [JM, DIM].

### Primary evaluations

We aimed to synthesize both process and final clinical outcome evaluations. While we did not limit inclusion to intervention studies, for the purpose of this review, the transition out of hospital after an elective surgery was conceptualized as a complex intervention and evaluations of the various aspects of the implementation of this process were included as process evaluations. A process evaluation explores the implementation of an intervention and can identify contextual factors that may be related to outcomes [23]. A final outcome assesses the extent to which an intervention is successful, [23] or in this context, the success of transitioning out of hospital after surgery.

A process evaluation, based on the UK Medical Research Council (MRC) framework, helps to understand the implementation, context and mechanisms of an intervention, [23] and the Ideal Transitions in Care (ITC) framework was used to help categorize processes within the MRC framework. The ITC framework includes 10 domains that the authors describe as analogous to the structural supports of a bridge that patients must cross from one care environment to another during the care transition process [24]. More specifically, “the ITC framework has been proposed as a method for analyzing failures and guiding new interventions in transitions of care, as well as creating process measures to monitor the quality of care transitions.” [25].

The American Geriatrics Society Health Care Systems Committee has highlighted the impact that transitions of care has on health outcomes, patient satisfaction and healthcare utilization [1]. The Institute for Healthcare Improvement (IHI) Triple Aim Framework posits that improvements across 3 similar areas are essential for transforming healthcare systems [26]. Therefore, outcome evaluations were categorized using the 3 domains of the IHI Triple Aim framework [26, 27] including 1) improving the individual experience of care, 2) improving the health of populations, and 3) reducing the cost of care for populations [26, 27]. The organization of outcome evaluations was informed by the IHI Guide to Measuring the Triple Aim [26] and previous work that has synthesized research using this framework [28].

### Data charting and analysis

A data charting form was used to capture relevant data from the included articles. Data from the first five articles and was charted and reviewed by EH and FD to reach agreement and finalize the form before proceeding to chart the data for the remaining articles. Data points extracted included: author and year, location, study design, sample size, patient characteristics (age, sex, frailty), surgical population characteristics (surgical specialty, surgical procedure), transitional care intervention (for effectiveness trials), whether the study used a theory or framework to conceptualize transitions in care, and whether the study reported that patient partners or knowledge users were engaged in the research. The ITC framework [24] and the Institute for Healthcare Improvement Triple Aim Framework [26] were used to categorize the processes and outcome evaluations, respectively.

## Results

### Search results

**Fig. 1:**
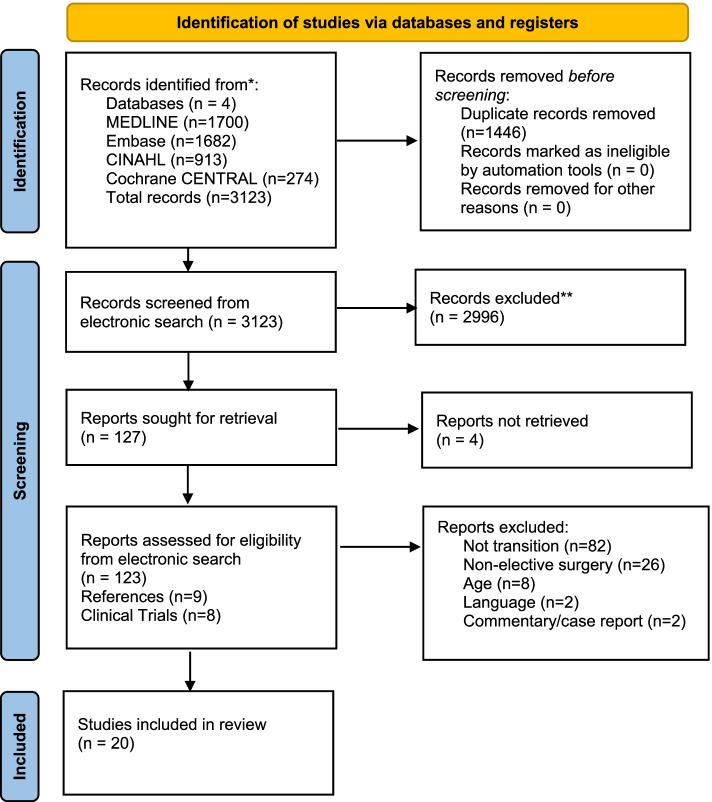
PRISMA 2020 flow diagram for new systematic reviews which included searches of databases and registers only

Our search identified 3123 citations. Of these, 127 abstracts were sought for full-text retrieval, 4 of which were not retrieved through multiple attempts, including trying to access the articles through multiple libraries and by contacting authors. This left 123 full-text articles to be reviewed in duplicate. During full-text article review, 103 articles were excluded (Additional File 3), leaving 20 articles for analysis. No additional articles were included after examining reference lists of the 20 included articles, relevant systematic and scoping reviews, or through clinical trial registries. A PRISMA flowchart detailing the screening process is provided in Fig. 1.

### Article characteristics

Characteristics of included articles are shown in Table 1. Additional File 4 provides descriptions of interventions in the nine articles that evaluated postoperative transitional care interventions. Sixteen articles were peer-reviewed manuscripts, and four were abstracts. Publication dates of included articles ranged from 2007 to 2020, with the majority conducted in the United States. Study designs included qualitative studies (*n* = 7), cohort studies (*n* = 5), randomized trials (*n* = 3), clinical prediction studies (*n* = 2), one quasi-experimental study (*n* = 1), one chart review study (*n* = 1) and one quality improvement project (*n* = 1).

**Table 1:** Article Characteristics

Author, Year, Country, Type	Objective	Study Design	Surgical Speciality; Procedure(s); Case Type(s)	Sample Size	Sex	Age
Missel et al., 2015, Denmark, Manuscript	To assess the effect of nurse-led systematic rehabilitation counselling performed before discharge to prevent concerns in the hospital-to-home gap in rehabilitation of lung cancer patients.	Quasi-experimental intervention study	Thoracic Surgery; Video-assisted thoracoscopic surgery, Thoracotomy; Elective	*n* = 120	Control group (59% female, 41% male); intervention group (43% female, 57% male)	Control group mean 69.6 (SD 9.4); intervention group mean 68 (SD 8.9)
Sawatsky et al., 2013; Canada; Manuscript	To describe and compare the outcomes of a Nurse Practitioner Follow-Up (NPFU) intervention with the standard model of post-discharge care for Coronary Artery Bypass Graft (CABG) patients.	Randomized clinical trial, two group, repeated measures design	Cardiac Surgery; Coronary Artery Bypass Graft; Elective	*N* = 200	81% male, 19% female	Mean 64.5
Young et al., 2013; Australia; Manuscript	To investigate the effectiveness of a centralized, nurse-delivered telephone-based service to improve care coordination and patient-reported outcomes after surgery for colorectal cancer.	Two-arm, parallel-group multi-centre randomized trial	General Surgery; Colorectal resections; Elective	*N* = 756	Control group (54.2% male); intervention group (56.8% male)	Control group mean 67 (SD 12.1); intervention group mean 68.6 (SD 12.2)
Middleton et al., 2004; Australia; Manuscript	To evaluate the short-term impact of nursing-led, coordinated care for patients after discharge following CEA.	Randomized Controlled Trial	Vascular Surgery; Re-vascularization surgery; Elective	*N* = 133	61.1% male, 38.9% female	Mean 70.4
Norcott et al., 2020; USA; Conference Poster Abstract	To evaluate whether a primary care visit before, rather than after, an elective surgery can potentially improve post-operative care transitions.	Retrospective observational cohort study	Unspecified; Unspecified; Elective	*N* = 1951	51% male, 49% female	Mean 77 (SD 5.6)
Czarnecki et al. 2019; Canada; Manuscript	To identify the causes of readmission after TAVR, determine whether transitional care factors were associated with a reduction in readmission and to identify other predictors that could be used to target quality improvement efforts.	Retrospective observational cohort study	Cardiac Surgery; Transcatheter Aortic Valve Replacement; 90.7% Elective, 9.3% Urgent	*N* = 937	55.7% male, 44.3% female	Median 83 (IQR 78–97)
Smucker et al., 2019; USA; Conference Poster Abstract	To identify predictors for unsuccessful postoperative transitions of care after major abdominal oncological operations.	Retrospective observational cohort study	General Surgery; Cancer resection Procedures (upper and lower GI, pancreatic, hepatobiliary); Elective	*N* = 240	55.4% men, 44.6% women	Median 67 (range 17 to 98)
McDonald et al., 2018; USA; Manuscript	To assess clinical outcomes for older adults undergoing elective abdominal surgery via a collaborative intervention by surgery, geriatrics, and anesthesia focused on perioperative health optimization.	Prospective observational cohort study	General Surgery; Colorectal, general, and hepato-pancreaticobiliary surgical procedures (specific procedures unspecified); Elective	*N* = 326	Control group (51.0% male, 49.0% female) intervention group (46.6% male, 54.4% female)	N
Shargall et al., 2016; Canada; Manuscript	To evaluate the Integrated Comprehensive Care (ICC) program, a novel health system integration initiative that coordinates home care and hospital-based clinical services for patients undergoing major thoracic surgery relative to traditional home care delivery.	Pilot retrospective cohort study	Thoracic Surgery; Open and Video-assisted thoracoscopic surgeries. Resections (wedge, multiple wedges, segmentectomy, lobectomy, bilobectomy, pneumonectomy, pleural, mediastinum); Elective	*N* = 686	Control group (58% male, 42% female); intervention group (48.5% male, 51.5% female)	Control group mean 63.8 (SD 0.78) intervention group mean 65.6 (0.71)
Xourafas et al., 2016; USA; Manuscript	To identify transitional care gaps, which could point out preventive perioperative clinical pathways to decrease post-pancreatectomy readmissions for nutritional causes.	Retrospective observational cohort study	General Surgery; Pancreatic resections; Elective	*N* = 172	non-readmitted group (51.7% female), readmitted group (67.7% female)	Non-re-admitted group median 66 (range 25 to 90); re-admitted group median 67 (range 32 to 83).
Weinberg et al., 2007; USA; Manuscript	To examine whether effective relational coordination better prepares caregivers to provide and manage care and examine the effects of caregiver preparation on patient clinical outcomes.	Prospective observational cohort study	Orthopaedic Surgery; Knee replacements; Elective	*N* = 222	38% male, 62% female	Mean 66.34 (SD 10.16)
Weinberg et al., 2007; USA; Manuscript	To investigate patients’ experience with coordination of their postsurgical care across multiple settings and the effects on key outcomes.	Prospective observational cohort study	Orthopaedic Surgery; Knee replacements; Elective	*n* = 222 patients *n* = 2 surgeons	38% male, a/62% female	Mean 66.34 (SD 10.16)
Quinlan et al., 2020; Canada; Conference Abstract	To design, implement and evaluate an adaptable smart phone or tablet design technology that would be offered to patients in follow up in addition to Interactive Voice Response technology, email and texting options, based on patient choice.	Quality Improvement Project	Cardiac Surgery; Transcatheter Aortic Valve Implants; 80% Elective, 20% Urgent	*N* = 265	Not reported	Mean 80
Brooke et al., 2019; USA; Manuscript	To characterize the extent to which information regarding a medically complex older patient’s functional status, cognitive status, social status, and emotional factors are mutually shared among PCPs and surgical providers during transitions of surgical care.	Qualitative interview study	General and Vascular Surgery; Procedures unspecified; Elective	*N* = 12 patients *N* = 35 Health-Care providers	83% male, 17% female [patients]	Mean 68.75 (SD 5.49) [patients]
Oksholm et al., 2018; Norway; Manuscript	To explore patients’ experiences of being transferred between hospitals after lung cancer surgery. The study aim was to improve the quality of transitional care.	Qualitative interview study	Thoracic Surgery; specific procedure unspecified; Elective	*N* = 14	6 men, 8 women	Mean 72 (range 56 to 87 years)
Wong et al., 2018; Canada; Manuscript	To minimize common risks associated with care transitions (such as early signs of heart failure exacerbation, complications with incisional sites, and medication confusion), and to promote optimal recovery through patient education and self-care support.	Pilot initiative using qualitative interviews	Cardiac Surgery; Transcatheter Aortic Valve Implants; 80% Elective, 20% Urgent	*N* = 77	40% male, 60% female	Median 84 (range 61 to 96)
Chan et al., 2017; USA; Conference Poster Abstract	To understand the geriatric surgical journey by identifying the personal and systemic factors associated with care transitions, readmissions, and care satisfaction, and to inform optimal care transitions for older adults experiencing elective surgery.	Qualitative interview study	Unspecified; Unspecified; Elective	*N* = 15	66% male, 44% female	mean 71 [range 65 to 88]
Thomsen et al., 2017; Denmark; Manuscript	To identify the perspectives of fast-track colorectal cancer surgery patients on challenges experienced in the transition from being a hospitalized patient with cancer to being a cancer survivor.	Qualitative participatory action research	General Surgery; Colorectal resections; Elective	*N* = 12	42% male, 58% female	Mean 72.4
Slager et al., 2017; USA; Manuscript	To characterize what, where, when and how surgical and primary care providers communicate clinical goals and expectations during transitions of care.	Qualitative interview study	General and Vascular Surgery; Procedures unspecified; Elective	25 patients, 17 surgical providers, and 16 Primary Care physicians	55% male, 45% female	Mean 68.75 (SD of 5.49) [patients]
Hughes et al., 2000; USA; Manuscript	To describe information needs of elderly postsurgical cancer patients.	Descriptive qualitative study	Oncological Surgery; Prostate, breast, gastrointestinal, lung, and head and neck cancer procedures (specific procedures unspecified); Elective	*N* = 148	57% male, 43% female	61% were 65 and older

Fifteen articles (75%) explored the postoperative transition from hospital to home [29–43] and five (25%) explored the postoperative transition from hospital to home or a new location including inpatient rehabilitation, skilled nursing facility, another hospital or hospice [44–48]. Two articles (9.5%) used theoretical frameworks to help inform a transitional care intervention including a theoretical framework based on motivational interviewing [43, 49] and Gittell’s theory of relational coordination [35, 50] and one article (4.8%) used the Nursing Model for Chronic Illness Management as a theoretical framework to inform their evaluation [42, 51]. Frailty was assessed and reported in two (9.5%) included articles using diagnostic codes from administrative data [44] and the Clinical Frailty Scale [48]. No articles reported that authors engaged patient partners or other knowledge users in the development of transitional care interventions or evaluations of the postoperative transitions in care.

### Processes evaluated – organized by the ideal transitions in care framework

Table 2 maps process evaluations to the domains in the Ideal Transitions in Care Framework, where applicable, and includes frequencies by domain. Additional File 5 provides descriptions of the process evaluations for all articles that included a process evaluation. Of the twenty included articles, 10 articles included process evaluations; some evaluated multiple processes.

**Table 2:** Process Evaluations

Author and year; study design	Discharge planning	Complete Communication of information	Availability, timeliness, clarity, and organization of information	Medication Safety	Educating patients to promote self-management	Enlisting help of social and community supports	Advance care planning	Coordinating care among team members	Monitoring and managing symptoms after discharge	Outpatient follow-up
Missel et al. 2015; Quasi-experimental intervention study	**X**				**X**	**X**				
Middleton et al. 2004; Randomized Controlled Trial					**X**					
Shargall et al. 2016; Pilot retrospective cohort study						**X**				
Xourafas et al. 2016; Observational cohort study	**X**	**X**			**X**					**X**
Weinberg et al. 2007; Prospective cohort study					**X**					**X**
Weinberg et al. 2007; Prospective cohort study								**X**		
Brooke et al. 2019; Qualitative interview study		**X**						**X**		
Wong et al. 2018; Pilot qualitative study				**X**	**X**				**X**	**X**
Slager et al. 2017; Qualitative interview study								**X**		
Hughes et al. 2000; Descriptive qualitative study					**X**					
**Total**	**2**	**2**	**0**	**1**	**6**	**2**	**0**	**3**	**1**	**3**

In the domain of ‘educating and promoting self-management’, two articles explored patient knowledge. One article evaluated patient knowledge of how and where to seek support and how to manage their care at home [43]. A second article evaluated patient knowledge of stroke risk factors and management of risk factors [31]. Two articles evaluated adherence regarding self-management, including adherence with discharge and care instructions once home [34] and adherence with tracking weight [48]. One article explored caregiver preparation to provide care after surgery, [35] and one article evaluated patient information needs once home after surgery [42].

In the domain of ‘coordinating care among team members’, three articles sought to understand care coordination from the perspective of key stakeholders. This included challenges of coordination between providers and between providers and patients, [36] concordance of information between patients, surgeons and primary care providers [38] and strategies to optimize communication between providers [41].

In the domain of ‘outpatient follow-up’, one article evaluated outpatient management as a transitional care variable used to predict readmission, [34] one article explored caregiver-reported interactions with formal providers [35] and one article evaluated patient-reported difficulty/ease of accessing their family doctor after surgery [48].

### Outcomes evaluated – organized by the IHI triple aim outcome domains

Outcome evaluations were spread relatively consistently across the three domains of the IHI Triple Aim framework as shown in Table 3. Nine articles reported population health evaluations, nine reported experiential evaluations and ten included cost evaluations. Two of the three randomized controlled trials included evaluations in all three domains [29, 30].

**Table 3:** Outcome Evaluations

Author/Study Design	Population Health Measures	Experience Measures	Cost Measures
Missel et al., 2015; Quasi-experimental intervention study	NA	**Patient experience:** patient experience regarding whether they felt supported with transitioning back to daily life, and patient rated physiological, psychological, social and existential outcomes using a questionnaire based on two validated questionnaires	NA
Sawatzky et al., 2013; Randomized clinical trial, two group, repeated measures design	**Quality of life**: measured using The Short Form-36 Health Survey; physical and mental component scores were also calculated. **Specific symptoms related to cardiac surgery recovery**: using the patient-reported Symptom Inventory and via interviews	**Patient satisfaction:** quality of service and the amount of help received was measured using the Client Satisfaction Questionnaire (CSQ-8).	**Utilization of care:** The Health Care Resources Utilization (HCRU) Questionnaire was developed by the researchers to operationalize the outcome of healthcare costs. This questionnaire simply elicited the self-reported number of participant contacts/visits with their primary care provider and cardiologist, as well as emergency department (ED) visits and hospital admissions at the 2 and 6-week post discharge interviews.
Young et al., 2013; Two-arm, parallel-group multi-center randomized trial	**Distress and quality of life:** Distress measured using the Distress Thermometer and quality of life measured using a PROM called the Functional Assessment of Cancer Therapy-Colorectal (FACT-C, specific to colorectal cancer).	**Patient experience:** patient experience of care coordination using a questionnaire; patient experience of cancer care coordination using the Supportive Care Needs Survey Short Form (SCNS-SF34); patient views of the intervention using interviews.	**Utilization of care:** postoperative neoadjuvant therapy, readmission, ED visits at 1 and 6 months using a questionnaire.
Middleton et al., 2004; Randomized controlled trial	**Stroke risk factors:** using blood pressure **Cholesterol level:** using a single question **Health status:** self-reported measured using a likert-scale **Physical activity:** self-reported measured using questionnaire **Smoking status:** self-reported using a single question **Changes to lifestyle to reduce risk of stroke:** self-reported using questionnaire	NA	NA
Norcott et al., 2020; Observational cohort study	NA	NA	**Utilization of care:** non-routine urgent postoperative care, defined as 30-day Emergency Department (ED), 30-day hospital readmissions, or either
Czarnecki et al., 2019; Retrospective observational cohort study	NA	NA	**Utilization of care:** all-cause readmission within 1 year of discharge using an administrative dataset held at the Institute of Clinical Evaluative Sciences
Smucker et al., 2019; Prediction study	NA	NA	**Utilization of care:** failure to discharge home, intermediate care in a Skilled Nursing Facility (SNF,) 90-day unplanned readmission
McDonald et al., 2018; Cohort study	**Complications**: obtained using International Classification of Diseases, Ninth Revision, Clinical Modification codes associated with the index surgical hospitalization obtained from billing data **Delirium:** measured using the Confusion Assessment Method criteria	NA	**Utilization of care:** length of stay (LOS), readmission rates (7-day and 30-day, all-cause inpatient readmission), and discharge disposition (home with self-care vs need for ongoing health services including home health, skilled nursing facility, or hospice) was obtained via medical records
Shargall et al., 2016; Pilot retrospective cohort study	**Mortality:** overall 60-day mortality and mortality for readmitted patients up to 60 days after readmission	**Patient satisfaction:** patient satisfaction of the Integrated Comprehensive Care Program using a satisfaction survey	**Utilization of care:** hospital length of stay, 60-day readmission rate, 60-day Emergency Room visits collected via the St. Joseph’s Healthcare Hamilton administrative dataset and the Local Health Integration Network-integrated dataset **Economic analysis:** cost of intervention reported only in intervention arm (no comparison of homecare costs was possible) based on homecare costs and comparison of inpatient costs between groups (case costing data, consisting of the average direct surgical and nonsurgical inpatient costs) according to the Ontario Case Costing Initiative methodology for 2011–2013 data
Xourafas et al., 2016; Observational cohort study	NA	NA	**Utilization of care:** 30-day readmission from dehydration or failure to thrive collected by chart review
Weinberg et al. 2007; Prospective cohort study	**Freedom from pain and functional status:** using questions from the PROM Western Ontario and McMaster University Osteoarthritis Index (WOMAC), a validated self-administered osteoarthritis instrument). **Mental health:** using the mental health component of the Short Form (36-item) Health Survey.	NA	NA
Weinberg et al., 2007; Prospective cohort study	**Freedom from pain and functional status:** using questions from the PROM Western Ontario and McMaster University Osteoarthritis Index (WOMAC), a validated self-administered osteoarthritis instrument). **Mental health:** using the mental health component of the Short Form (36-item) Health Survey.	**Patient satisfaction:** patient satisfaction with overall care during the post-discharge period with one question asking patients to rate their care using a likert scale	NA
Quinlan et al., 2020; Quality Improvement project	**Quality of life**: measured using the Short Form (12-item) Health Survey.	**Patient satisfaction:** measured using a question as to whether patients thought their contact method was a good way for follow-up after hospital discharge	**Gaps in care:** gaps in transitional care were identified by the Advanced Practice Nurse
Brooke et al., 2019; Qualitative interview study	NA	NA	NA
Oksholm et al., 2018; Qualitative interview study	NA	**Patient experience:** overall patient experience using qualitative interviews and the hermeneutic analysis method	NA
Wong et al., 2018; Pilot qualitative study	**Cardiac symptoms:** described by patients over the phone and categorized using New York Heart Association (NYHA) **Perceived Health:** measured using a 1–10 likert scale **Self-rated Pain:** using a 1–10 likert scale **Weight change:** patient-reported changes in weight using a scale **Depression and Anxiety:** measured using a patient screening tool **Participating in physical activity:** patient-reported over the telephone	NA	**Utilization of care:** 30-day readmission
Chan et al., 2017; Qualitative interview study	NA	**Patient experience:** overall patient experience using interviews in order to identify the personal and systemic factors associated with care transitions, readmissions, and care satisfaction, and to inform optimal care transitions	NA
Thomsen et al., 2017; Qualitative participatory action research	NA	**Patient experience:** overall patient experience and perspectives using qualitative interviews using a participatory action research methodology	NA
Slager et al., 2017; Qualitative interview study	NA	NA	NA
Hughes et al., 2000; Descriptive qualitative study	NA	NA	NA

#### Population health

Of the nine articles reporting health outcomes, seven articles evaluated health status or quality of life, all using patient-reported outcome measures [29–31, 35–37, 48]. Symptoms were evaluated in six articles, including pain in three articles [35, 36, 48], depression and anxiety in one article [48], and cardiac symptoms in two articles [29, 48].

Four articles evaluated risk factors for repeat events or complications. Three articles included physiological or anthropometric evaluations that were specific to cardiac or vascular disease including weight change, [48] blood pressure [31] and cholesterol levels [31]. One article evaluated postoperative complications [46].

Three articles evaluated behaviour change. Two articles evaluated participation in physical activity after surgery [31, 48] and one article evaluated changes in lifestyle to reduce risk of stroke [31].

Two articles evaluated function using a patient-reported outcome measure [35, 36]. Delirium was evaluated in one article [46] and mortality was evaluated in one article [33].

#### Experience

Nine articles evaluated experiential outcomes. Of these, four articles examined patient satisfaction using patient-reported experience measures [29, 33, 36, 37]. Two qualitative studies examined specific aspects of the patient experience, including the psychological and social experience [43] and the experience of care coordination [30]. Three articles explored overall patient experiences with transitions in care using qualitative methodologies [39, 40, 47]. Of the three qualitative articles investigating the overall experiences of patients, one explored the overall patient experience of being transferred between hospitals after lung cancer surgery, [47] a second explored the challenges experienced during the transition from a hospitalized patient after colorectal surgery to a cancer survivor, [39] and a third evaluated the experience of the geriatric surgical journey including relevant personal and systemic factors to inform optimal care transitions for older adults [40].

#### Cost

Ten articles included evaluations in the cost domain. Utilization of health services was the most common construct evaluated in this domain. Nine articles evaluated readmission rates [29, 30, 32–34, 44–46, 48] four articles evaluated visits to the Emergency Department (ED), [29, 30, 32, 33] two articles evaluated hospital length of stay, [33, 46] two articles evaluated discharge disposition [45, 46] and one article evaluated the number of contacts with the primary care provider and cardiologist after surgery [29]. One article evaluated the cost of the intervention based on homecare costs and comparison of inpatient costs [33]. One article evaluated gaps in transitional care [37]. No articles included a cost per capita, cost benefit, or cost utility analysis.

## Discussion

This scoping review identified and described the processes and outcomes that have been used in research to evaluate postoperative transitions in care for older adults. The results demonstrate heterogeneity in the evaluations that have been used to date. Ten of twenty included articles evaluated postoperative transition in care processes, most commonly evaluated using ITC categories of ‘educating and promoting self-management’, ‘coordinating care among team members’ and ‘outpatient follow-up’. Eighteen of twenty included articles evaluated transitions using final outcomes, distributed relatively evenly across the three domains of the IHI Triple Aim Framework. As a result of the inconsistency in evaluations, there remains a need to define a core outcome set, [52] informed by patients and caregivers, to produce meaningful and consistent evaluations in postoperative transitions in care for older adults.

The findings from this review are similar to that of Leibzeit’s review of transitional care interventions for older adults leaving hospital following a general medicine admission [16]. Leibzeit [16] and colleagues found that the most common outcomes evaluated were readmission, mortality and quality of life. The reviewers identified the most common components of transitional care interventions included ‘care continuity and coordination’, ‘medication management’, ‘symptom recognition’ and ‘self-management’. The current review adds to this evidence base by indicating similar processes and outcomes were evaluated in postoperative transitions in care for older adults.

Further, while a systematic review of transitional care interventions for surgical patients has been conducted, [53] and found that transitional care interventions may reduce readmission rates, the search was limited by outcome (90-day readmission) and was not specific to older adults. The current scoping review is the first knowledge synthesis study, to our knowledge, that has been specific to older adults and the transition in care leaving hospital after surgery. It adds to the evidence on transitional care interventions by depicting the variability in how postoperative transitions in care for older adults are being evaluated.

Transitions in care are a period of increased risk for older adults due to their complex needs. Improving the quality and outcomes of transitional care after surgery is urgently required [15]. However, consistent targets are needed to define a high quality or successful transition in care in order to maximize the impact of research and evaluation on patients, caregivers and the healthcare system. This review demonstrates that there is inconsistency in how transitions in care have been evaluated to date in the literature. Development of a core outcome set, or an agreed-upon set of outcomes to be evaluated to ensure standardization, [52] for postoperative transitions in care for older adults is an important step that would help to increase consistency of outcomes evaluated. Core outcome sets that include meaningful patient, caregiver and other expert engagement have several potential benefits, including: facilitating the opportunity for systematic review and meta-analysis, reduced risk of reporting bias in future trials, and consensus on what to evaluate based on what is most important to patients and caregivers [54, 55].

None of the articles identified in this review included knowledge users, such as patients and caregivers, during the process of selecting process and outcome evaluations. Engagement of knowledge users in the selection of evaluation approaches may help to prioritize what evaluations to include in future research on postoperative transitions in care for older adults [56]. Engagement of patients and caregivers often lead to selection of different evaluations within research [57]. For example, one recent study explored patient and caregiver priorities for patients leaving hospital following non-surgical admission and discovered that having more publicly funded and timely access to homecare were among the top priorities [58]. Yet, accessibility of health services was only evaluated in one study in the present scoping review, which evaluated uptake of homecare services [33]. This provides one example of when patient priorities and what is being evaluated in the literature do not currently align. Leibzeit [16] similarly noted that caregiver engagement and education are currently missing components in transitional care interventions for older adults and that these important aspects must be considered in future research. Further, other researchers have identified the need to improve postoperative transitions when patients require post-acute care including admissions to skilled nursing facilities and inpatient rehabilitation facilities [59]. Therefore, it would be important to determine from patients whether priorities are different based on discharge location following surgery.

Despite the variability found in evaluations, there is encouraging data from this review. Process evaluations are being used in the transitions in care literature for older adults having surgery including process evaluations that align with the domains of the Ideal Transitions in Care Framework which helps to ensure a safe and successful transition out of hospital. Process evaluations are essential for understanding key aspects of an intervention including the implementation and context of an intervention which was evaluated by both quantitative and qualitive research included in this review. Patient-reported outcomes were used consistently in the population health domain of the Triple Aim Framework to evaluate function, health status, and quality of life. Additionally, several articles explored patient experience. These are important findings because outcomes such as function, independence and patient experience are patient-centered and tend to be valued by older adults [60, 61]. It is imperative to evaluate these outcomes as patient priorities tend to differ from those of clinicians and the healthcare system [61].

Notably, the majority of included articles that evaluated outcomes in the in the ‘costs’ domain of the IHI Triple Aim framework focused on healthcare utilization (e.g. hospital readmissions, Emergency Department visits, length-of stay and non-home discharge), but cost per capita, cost-benefit or cost-utility analyses were not included in any of the included articles. Given the tremendous costs associated with adverse transitions in care for older adults [3], this highlights an important gap for future research on transitions in care.

The findings of this scoping review point to a number of areas for future research. For example, additional future qualitative research is needed to understand the experiences of patients transitioning out of hospital after surgery with shared experiences (e.g., surgical procedure, transition location). This research is required to understand whether transitional care interventions and their respective evaluations need to be tailored based on the type of surgery, patient population or transition setting. While it is not always the goal of qualitative research to identify generalizable or transferrable findings, these findings are important as The World Health Organization (WHO) states that patient perspectives, experiences and needs are an integral part of transitions in care [62]. Further, these findings align with the ‘Experience of Care’ domain of the IHI Triple Aim Framework and can allow researchers and clinicians to better understand patient experiences to provide meaningful improvements in experiential outcomes. Additional research to identify the highest priorities of older adults transitioning out of hospital after surgery could also help to inform the development and evaluation of transitional care interventions. As only two articles identified frailty among their study participants, there is a need to explore the preferences and needs of these vulnerable older adults to develop interventions specifically for this population. Finally, of the twenty included articles in this scoping review, eight included interventions, and only three were randomized controlled trials. There is a need to develop and robustly evaluate transitional care interventions for older adults having surgery that will result in meaningful improvements for patients, caregivers and the healthcare system.

### Limitations

There are limitations to consider with our review. First, only articles that were published in English or French were included. While our search included reviewing reference lists and clinical trial registries, other grey literature was not examined. Further, urgent surgeries such as hip fractures, which are common among an older population, were excluded from this review as the focus was on elective surgery. Future research should consider exploring postoperative transitions in care for older adults requiring urgent surgery.

## Conclusions

Current process and outcome evaluations of postoperative transitions in care for older adults are heterogeneous. The most common outcomes evaluated were utilization of services, including readmission and Emergency Department visits, experiential outcomes and quality of life. Process evaluations most frequently focused on educating and promoting self-management, care coordination and outpatient follow-up. This review provides evidence on how transitions in care after surgery have been evaluated in the literature to date, which provides important information on research gaps and an opportunity for future research to determine if the evaluations used in the literature align with what is important to key stakeholders. No articles engaged patient and caregiver knowledge users in decisions about the approach to evaluations used. Future research should identify what processes and outcomes are important to older adults and their caregivers during postoperative transitions in care.

## Supplementary Information

**Additional file 1.**

**Additional file 2.**

**Additional file 3.**

**Additional file 4.**

**Additional file 5.**

## Data Availability

All data generated or analysed during this study are included in this published article [and its supplementary information files]. Any additional data are available from the corresponding author on reasonable request.

## Abbreviations

IHI: Institute for Healthcare Improvement
ITC: Ideal Transitions in Care
MRC: Medical Research Council
WHO: World Health Organization
