# Supplementary material for: Evaluations of postoperative transitions in care for older adults: a scoping review
Source: BMC Geriatr. 2022 Apr 15;22:329. doi: 10.1186/s12877-022-02989-6 (PMC9013054; doi:10.1186/s12877-022-02989-6)
Supplement: Supplementary file 2 — Additional file 2. [file 12877_2022_2989_MOESM2_ESM.docx]

**Additional File 2. Peer Review of Electronic Search Strategies (PRESS) process**

**Search 1 – October 3, 2019**

**Database: Ovid MEDLINE(R) ALL <1946 to October 03, 2019>
Search Strategy:**
--------------------------------------------------------------------------------
1     Transitional Care/ (587)
2     (transition* adj3 care).tw. (5936)
3     (transition* and care).ti. (3551)
4     transition* care.kw. (784)
5     (transition* adj3 home).tw. (716)
6     "Continuity of Patient Care"/ (18368)
7     (continu* adj3 care).tw. (20156)
8     "coordination of care".tw. (1690)
9     (handover* or handoff*).tw,kf. (2627)
10     "hospital to home".tw. (2637)
11     ((discharge or postdischarge or post discharge) adj3 (training or planning or program* or support or intervention* or instruction* or teaching or education)).tw. (6704)
12     *Patient Education as Topic/ or patient education.ti,kw. (39974)
13     (education* adj2 (program* or intervention*)).tw. (57159)
14     home care services/ or Home Nursing/ (40025)
15     or/12-14 (131321)
16     *patient readmission/ or (discharg* or readmission*).tw. (264789)
17     15 and 16 (4983)
18     1 or 2 or 3 or 4 or 5 or 6 or 7 or 8 or 9 or 10 or 11 or 17 (54095)
19     "Aged, 80 and over"/ (867258)
20     (elderly or seniors or geriatric or older person$ or older people or older adult$ or older women or older men or octogenarian$ or septuagenarian$ or centenarian$ or nonagenarian$).tw,kw. (367468)
21     older patient*.tw. (37644)
22     (("sixty-five" or "65" or seventy or "70" or eighty or "80") adj2 (aged or age or over or older)).tw,kw. (125840)
23     aged/ and middle aged/ (2308434)
24     or/19-23 (2725290)
25     exp Surgical Procedures, Operative/ or Postoperative Period/ or Preoperative Period/ or Preoperative Care/ or Postoperative Complications/ (3110523)
26     (postoper* or post oper* or postsurg* or post surg* or preoper* or pre oper*).ti,kw. (141533)
27     exp Hip Fractures/su (10792)
28     ((postoper* or post oper* or postsurg* or post surg* or preoper* or pre oper*) adj3 (patient* or care)).tw. (121755)
29     surg* patient*.tw. (40644)
30     ((after or following or underwent) adj3 (surg* or fracture* or resection* or operation*)).tw. (540715)
31     or/25-30 (3386189)
**32     18 and 24 and 31 (1569**)

**Database: Embase Classic+Embase <1947 to 2019 October 03>
Search Strategy:**--------------------------------------------------------------------------------
1     transitional care/ (2433)
2     (transition* adj3 care).tw. (10427)
3     (transition* and care).ti. (5191)
4     (transition* adj3 home).tw. (1141)
5     (continuum adj3 care).tw. (5838)
6     "hospital to home".tw. (3949)
7     (care adj2 coordination).tw. (7289)
8     (care adj3 continuity).tw. (10448)
9     (handover* or handoff*).tw. (4041)
10     ((discharge or postdischarge or post discharge) adj3 (training or planning or program* or support or intervention* or instruction* or teaching or education)).tw. (10754)
11     *patient education/ or patient education.ti. (29734)
12     (education* adj2 (program* or intervention*)).tw. (78848)
13     home visit/ or home care/ (63571)
14     or/11-13 (167116)
15     *hospital readmission/ or (discharg* or readmission*).tw. (443002)
16     14 and 15 (8574)
17     1 or 2 or 3 or 4 or 5 or 6 or 7 or 8 or 9 or 10 or 16 (56690)
18     aged/ (3028714)
19     elderly care/ (39978)
20     (elderly or seniors or geriatric or older person$ or older people or older adult$ or older women or older men or octogenarian$ or septuagenarian$ or centenarian$ or nonagenarian$).tw. (515046)
21     older patient*.tw. (59218)
22     (("sixty-five" or "65" or seventy or "70" or eighty or "80") adj2 (aged or age or over or older)).tw. (211413)
23     or/18-22 (3286179)
24     surgical patient/ (47585)
25     exp *surgery/ (2444314)
26     (postoper* or post oper* or postsurg* or post surg* or preoper* or pre oper*).ti. (174690)
27     postoperative care/ or postoperative period/ or postoperative complication/ (605187)
28     perioperative period/ or preoperative period/ (95869)
29     ((after or following or underwent) adj3 (surg* or fracture* or resection* or operation*)).tw. (804493)
30     ((postoper* or post oper* or postsurg* or post surg* or preoper* or pre oper*) adj3 (patient* or care)).tw. (186853)
31     hip fracture/su [Surgery] (5436)
32     or/24-31 (3193103)
**33     17 and 23 and 32 (1435)**

**Database: EBM Reviews - Cochrane Central Register of Controlled Trials <August 2019>
Search Strategy:**--------------------------------------------------------------------------------
1     Transitional Care/ (38)
2     (transition* adj3 care).tw. (851)
3     (transition* and care).ti. (322)
4     transition* care.kw. (97)
5     (transition* adj3 home).tw. (239)
6     "Continuity of Patient Care"/ (578)
7     (continu* adj3 care).tw. (3023)
8     "coordination of care".tw. (138)
9     (handover* or handoff*).tw. (156)
10     "hospital to home".tw. (593)
11     ((discharge or postdischarge or post discharge) adj3 (training or planning or program* or support or intervention* or instruction* or teaching or education)).tw. (2509)
12     *Patient Education as Topic/ or patient education.ti,kw. (3223)
13     (education* adj2 (program* or intervention*)).tw. (14861)
14     home care services/ or Home Nursing/ (1949)
15     or/12-14 (19145)
16     *patient readmission/ or (discharg* or readmission*).tw. (34146)
17     15 and 16 (1169)
18     1 or 2 or 3 or 4 or 5 or 6 or 7 or 8 or 9 or 10 or 11 or 17 (7917)
19     "Aged, 80 and over"/ (50791)
20     (elderly or seniors or geriatric or older person$ or older people or older adult$ or older women or older men or octogenarian$ or septuagenarian$ or centenarian$ or nonagenarian$).tw,kw. (60217)
21     older patient*.tw. (4484)
22     (("sixty-five" or "65" or seventy or "70" or eighty or "80") adj2 (aged or age or over or older)).tw,kw. (38734)
23     aged/ and middle aged/ (176456)
24     or/19-23 (256725)
25     exp Surgical Procedures, Operative/ or Postoperative Period/ or Preoperative Period/ or Preoperative Care/ or Postoperative Complications/ (114398)
26     (postoper* or post oper* or postsurg* or post surg* or preoper* or pre oper*).ti,kw. (48209)
27     exp Hip Fractures/su (2)
28     ((postoper* or post oper* or postsurg* or post surg* or preoper* or pre oper*) adj3 (patient* or care)).tw. (29820)
29     surg* patient*.tw. (15321)
30     ((after or following or underwent) adj3 (surg* or fracture* or resection* or operation*)).tw. (8327)
31     or/25-30 (169877)
**32     18 and 24 and 31 (229)**

**CINAHL**

| **#** | **Query** | **Results** |
| --- | --- | --- |
| **S35** | **S5 AND S13 AND S34** | **803** |
| S34 | S14 OR S15 OR S16 OR S17 OR S18 OR S19 OR S20 OR S21 OR S22 OR S23 OR S24 OR S33 | 35,425 |
| S33 | S29 AND S32 | 4,891 |
| S32 | S30 OR S31 | 77,486 |
| S31 | TI ( (discharg* or readmission*) ) OR AB ( (discharg* or readmission*) ) | 73,461 |
| S30 | (MH "Readmission") | 11,715 |
| S29 | S25 OR S26 OR S27 OR S28 | 93,072 |
| S28 | (MH "Home Nursing") OR (MH "Home Health Care+") | 45,555 |
| S27 | TI ( (education N2 (program or intervention*)) ) OR AB ( (education N2 (program or intervention*)) ) | 23,364 |
| S26 | TI patient education | 3,740 |
| S25 | (MH "Patient Discharge Education") OR (MM "Patient Education") | 25,589 |
| S24 | TI "coordination of care" OR AB "coordination of care" | 1,143 |
| S23 | TI ( (handover* or handoff*) ) OR AB ( (handover* or handoff*) ) | 2,069 |
| S22 | TI ( ((discharge or postdischarge or post discharge) N3 (training or planning or program* or support or intervention* or instruction* or teaching or education)) ) OR AB ( ((discharge or postdischarge or post discharge) N3 (training or planning or program* or support or intervention* or instruction* or teaching or education)) ) | 5,595 |
| S21 | TI "hospital to home" OR AB "hospital to home" | 1,700 |
| S20 | TI (continuum N3 care) OR AB (continuum N3 care) | 3,228 |
| S19 | (MH "Continuity of Patient Care") | 12,982 |
| S18 | TI (care N3 continuity) OR AB (care N3 continuity) | 5,108 |
| S17 | TI (transition* N3 home) OR AB (transition* N3 home) | 1,151 |
| S16 | TI (transition* and care) | 3,481 |
| S15 | TI (transition* N3 care) OR AB (transition* N3 care) | 5,539 |
| S14 | (MH "Transitional Care") | 1,260 |
| S13 | S6 OR S7 OR S8 OR S9 OR S10 OR S11 OR S12 | 651,314 |
| S12 | (MH "Surgery, Operative+") | 577,570 |
| S11 | (MH "Hip Fractures+/SU") | 3,682 |
| S10 | TI ( ((after or following or underwent) N3 (surg* or fracture* or resection* or operation*)) ) OR AB ( ((after or following or underwent) N3 (surg* or fracture* or resection* or operation*)) ) | 96,294 |
| S9 | TI surg* patient* OR AB surg* patient* | 12,834 |
| S8 | TI ( ((postoper* or post oper* or postsurg* or post surg* or preoper* or pre oper*) N3 (patient* or care)) ) OR AB ( ((postoper* or post oper* or postsurg* or post surg* or preoper* or pre oper*) N3 (patient* or care)) ) | 31,282 |
| S7 | TI (postoper* or post oper* or postsurg* or post surg* or preoper* or pre oper*) | 31,582 |
| S6 | (MH "Postoperative Period") OR (MH "Postoperative Complications+") OR (MH "Intraoperative Period") OR (MH "Preoperative Period+") | 116,755 |
| S5 | S1 OR S2 OR S3 OR S4 | 698,186 |
| S4 | TI ( (("sixty-five" or "65" or seventy or "70" or eighty or "80") N2 (aged or age or over or older)) ) OR AB ( (("sixty-five" or "65" or seventy or "70" or eighty or "80") N2 (aged or age or over or older)) ) | 39,316 |
| S3 | TI older patient* OR AB older patient* | 13,845 |
| S2 | TI ( (elderly or seniors or geriatric or older person* or older people or older adult* or older women or older men or octogenarian* or septuagenarian* or centenarian* or nonagenarian*) ) OR AB ( (elderly or seniors or geriatric or older person* or older people or older adult* or older women or older men or octogenarian* or septuagenarian* or centenarian* or nonagenarian*) ) | 180,068 |
| S1 | (MH "Aged, 80 and Over+") OR (MH "Health Services for the Aged") or ((MH "Middle Age") AND (MH "Aged+")) | 578,706 |

**Search 2 – Updated on April 6, 2021**

Embase Classic+Embase <1947 to 2021 April 06>

Ovid MEDLINE(R) ALL <1946 to April 06, 2021>

EBM Reviews - Cochrane Central Register of Controlled Trials <March 2021>

Medline: 131
Embase: 247
Cochrane: 45
CinahlComplete: 110
Total: 533
After Duplicates: 436
Total Duplicates:  97

1 Transitional Care/ 4484

2 (transition* adj3 care).tw. 21245

3 (transition* and care).ti. 10918

4 transition* care.kw. 1987

5 (transition* adj3 home).tw. 2584

6 "Continuity of Patient Care"/ 301687

7 (continu* adj3 care).tw. 59936

8 "coordination of care".tw. 5057

9 (handover* or handoff*).tw,kf. 8125

10 "hospital to home".tw. 8196

11 ((discharge or postdischarge or post discharge) adj3 (training or planning or program* or support or intervention* or instruction* or teaching or education)).tw. 23057

12 *Patient Education as Topic/ or patient education.ti,kw. 78608

13 (education* adj2 (program* or intervention*)).tw. 170764

14 home care services/ or Home Nursing/ 107247

15 or/12-14 343863

16 *patient readmission/ or (discharg* or readmission*).tw. 853906

17 15 and 16 15981

18 or/1-11,17 413517

19 "Aged, 80 and over"/ 1119952

20 (elderly or seniors or geriatric or older person* or older people or older adult* or older women or older men or octogenarian* or septuagenarian* or centenarian* or nonagenarian*).tw,kw. 1072610

21 older patient*.tw. 115899

22 (("sixty-five" or "65" or seventy or "70" or eighty or "80") adj2 (aged or age or over or older)).tw,kw. 432480

23 aged/ and middle aged/ 3458993

24 or/19-23 4791834

25 exp Surgical Procedures, Operative/ or Postoperative Period/ or Preoperative Period/ or Preoperative Care/ or Postoperative Complications/ 8990764

26 (postoper* or post oper* or postsurg* or post surg* or preoper* or pre oper*).ti,kw. 438328

27 exp Hip Fractures/su 22783

28 ((postoper* or post oper* or postsurg* or post surg* or preoper* or pre oper*) adj3 (patient* or care)).tw. 379521

29 surg* patient*.tw. 134297

30 ((after or following or underwent) adj3 (surg* or fracture* or resection* or operation*)).tw. 1497501

31 or/25-30 9595419

32 18 and 24 and 31 6241

33 32 use medall 1821

34 (20191* or 2020* or 2021*).dt. 2242815

**35 33 and 34 131 Medline**

36 transitional care/ 4484

37 (transition* adj3 care).tw. 21245

38 (transition* and care).ti. 10918

39 (transition* adj3 home).tw. 2584

40 (continuum adj3 care).tw. 12129

41 "hospital to home".tw. 8196

42 (care adj2 coordination).tw. 15438

43 (care adj3 continuity).tw. 21567

44 (handover* or handoff*).tw. 8058

45 ((discharge or postdischarge or post discharge) adj3 (training or planning or program* or support or intervention* or instruction* or teaching or education)).tw. 23057

46 *patient education/ or patient education.ti. 73161

47 (education* adj2 (program* or intervention*)).tw. 170764

48 home visit/ or home care/ 106397

49 or/46-48 338500

50 *hospital readmission/ or (discharg* or readmission*).tw. 854079

51 49 and 50 15739

52 or/36-45,51 116331

53 aged/ 6710629

54 elderly care/ 41223

55 (elderly or seniors or geriatric or older person* or older people or older adult* or older women or older men or octogenarian* or septuagenarian* or centenarian* or nonagenarian*).tw. 1054922

56 older patient*.tw. 115899

57 (("sixty-five" or "65" or seventy or "70" or eighty or "80") adj2 (aged or age or over or older)).tw. 430951

58 or/53-57 7256283

59 surgical patient/ 52749

60 exp *surgery/ 2700298

61 (postoper* or post oper* or postsurg* or post surg* or preoper* or pre oper*).ti. 374008

62 postoperative care/ or postoperative period/ or postoperative complication/ 1150875

63 perioperative period/ or preoperative period/ 121157

64 ((after or following or underwent) adj3 (surg* or fracture* or resection* or operation*)).tw. 1497501

65 ((postoper* or post oper* or postsurg* or post surg* or preoper* or pre oper*) adj3 (patient* or care)).tw. 379521

66 hip fracture/su 13076

67 or/59-66 4668471

68 52 and 58 and 67 2640

69 68 use emczd 1668

70 (20191* or 2020* or 2021*).dc. 3270286

**71 69 and 70 247 Embase**

72 Transitional Care/ 4484

73 (transition* adj3 care).tw. 21245

74 (transition* and care).ti. 10918

75 transition* care.kw. 1987

76 (transition* adj3 home).tw. 2584

77 "Continuity of Patient Care"/ 301687

78 (continu* adj3 care).tw. 59936

79 "coordination of care".tw. 5057

80 (handover* or handoff*).tw. 8058

81 "hospital to home".tw. 8196

82 ((discharge or postdischarge or post discharge) adj3 (training or planning or program* or support or intervention* or instruction* or teaching or education)).tw. 23057

83 *Patient Education as Topic/ or patient education.ti,kw. 78608

84 (education* adj2 (program* or intervention*)).tw. 170764

85 home care services/ or Home Nursing/ 107247

86 or/83-85 343863

87 *patient readmission/ or (discharg* or readmission*).tw. 853906

88 86 and 87 15981

89 or/72-82,88 413471

90 "Aged, 80 and over"/ 1119952

91 (elderly or seniors or geriatric or older person* or older people or older adult* or older women or older men or octogenarian* or septuagenarian* or centenarian* or nonagenarian*).tw,kw. 1072610

92 older patient*.tw. 115899

93 (("sixty-five" or "65" or seventy or "70" or eighty or "80") adj2 (aged or age or over or older)).tw,kw. 432480

94 aged/ and middle aged/ 3458993

95 or/90-94 4791834

96 exp Surgical Procedures, Operative/ or Postoperative Period/ or Preoperative Period/ or Preoperative Care/ or Postoperative Complications/ 8990764

97 (postoper* or post oper* or postsurg* or post surg* or preoper* or pre oper*).ti,kw. 438328

98 exp Hip Fractures/su 22783

99 ((postoper* or post oper* or postsurg* or post surg* or preoper* or pre oper*) adj3 (patient* or care)).tw. 379521

100 surg* patient*.tw. 134297

101 ((after or following or underwent) adj3 (surg* or fracture* or resection* or operation*)).tw. 1497501

102 or/96-101 9595419

103 89 and 95 and 102 6240

104 103 use cctr 275

**105 limit 104 to yr="2019 -Current" 45 Cochrane**

106 35 or 71 or 105 423

107 remove duplicates from 106 349

108 107 use medall 130

109 107 use emczd 188

110 107 use cctr 31 Top of Form

**CinahlComplete**

|  | Wednesday, April 07, 2021 1:25:04 PM |
| --- | --- |

| **#** | **Query** | **Results** |
| --- | --- | --- |
| S1 | (MH "Aged, 80 and Over+") OR (MH "Health Services for the Aged") or ((MH "Middle Age") AND (MH "Aged+")) | 675,447 |
| S2 | TI ( (elderly or seniors or geriatric or older person* or older people or older adult* or older women or older men or octogenarian* or septuagenarian* or centenarian* or nonagenarian*) ) OR AB ( (elderly or seniors or geriatric or older person* or older people or older adult* or older women or older men or octogenarian* or septuagenarian* or centenarian* or nonagenarian*) ) | 217,254 |
| S3 | TI ( (elderly or seniors or geriatric or older person* or older people or older adult* or older women or older men or octogenarian* or septuagenarian* or centenarian* or nonagenarian*) ) OR AB ( (elderly or seniors or geriatric or older person* or older people or older adult* or older women or older men or octogenarian* or septuagenarian* or centenarian* or nonagenarian*) ) | 217,254 |
| S4 | TI ( (("sixty-five" or "65" or seventy or "70" or eighty or "80") N2 (aged or age or over or older)) ) OR AB ( (("sixty-five" or "65" or seventy or "70" or eighty or "80") N2 (aged or age or over or older)) ) | 48,342 |
| S5 | S1 OR S2 OR S3 OR S4 | 816,031 |
| S6 | (MH "Postoperative Period") OR (MH "Postoperative Complications+") OR (MH "Intraoperative Period") OR (MH "Preoperative Period+") | 141,417 |
| S7 | TI (postoper* or post oper* or postsurg* or post surg* or preoper* or pre oper*) | 38,428 |
| S8 | TI ( ((postoper* or post oper* or postsurg* or post surg* or preoper* or pre oper*) N3 (patient* or care)) ) OR AB ( ((postoper* or post oper* or postsurg* or post surg* or preoper* or pre oper*) N3 (patient* or care)) ) | 39,525 |
| S9 | TI surg* patient* OR AB surg* patient* | 15,611 |
| S10 | TI ( ((after or following or underwent) N3 (surg* or fracture* or resection* or operation*)) ) OR AB ( ((after or following or underwent) N3 (surg* or fracture* or resection* or operation*)) ) | 120,938 |
| S11 | (MH "Hip Fractures+/SU") | 4,495 |
| S12 | (MH "Surgery, Operative+") | 686,731 |
| S13 | S6 OR S7 OR S8 OR S9 OR S10 OR S11 OR S12 | 776,855 |
| S14 | (MH "Transitional Care") | 1,997 |
| S15 | TI (transition* N3 care) OR AB (transition* N3 care) | 7,030 |
| S16 | TI (transition* and care) | 4,215 |
| S17 | TI (transition* N3 home) OR AB (transition* N3 home) | 1,419 |
| S18 | TI (care N3 continuity) OR AB (care N3 continuity) | 6,322 |
| S19 | (MH "Continuity of Patient Care") | 15,349 |
| S20 | TI (continuum N3 care) OR AB (continuum N3 care) | 3,889 |
| S21 | TI "hospital to home" OR AB "hospital to home" | 2,009 |
| S22 | TI ( ((discharge or postdischarge or post discharge) N3 (training or planning or program* or support or intervention* or instruction* or teaching or education)) ) OR AB ( ((discharge or postdischarge or post discharge) N3 (training or planning or program* or support or intervention* or instruction* or teaching or education)) ) | 6,779 |
| S23 | TI ( (handover* or handoff*) ) OR AB ( (handover* or handoff*) ) | 2,500 |
| S24 | TI "coordination of care" OR AB "coordination of care" | 1,356 |
| S25 | (MH "Patient Discharge Education") OR (MM "Patient Education") | 28,489 |
| S26 | TI patient education | 4,116 |
| S27 | TI ( (education N2 (program or intervention*)) ) OR AB ( (education N2 (program or intervention*)) ) | 28,421 |
| S28 | (MH "Home Nursing") OR (MH "Home Health Care+") | 51,073 |
| S29 | S25 OR S26 OR S27 OR S28 | 106,255 |
| S30 | (MH "Readmission" | 14,683 |
| S31 | TI ( (discharg* or readmission*) ) OR AB ( (discharg* or readmission*) ) | 91,539 |
| S32 | S30 OR S31 | 96,234 |
| S33 | S29 AND S32 | 5,745 |
| S34 | S14 OR S15 OR S16 OR S17 OR S18 OR S19 OR S20 OR S21 OR S22 OR S23 OR S24 OR S33 | 42,847 |
| S35 | S5 AND S13 AND S34 | 956 |
| S36 | EM 20191001- | 537,862 |
| S37 | S35 AND S36 | 110 |
|  |  |  |

Bottom of Form
