# Supplementary material for: Evaluations of postoperative transitions in care for older adults: a scoping review
Source: BMC Geriatr. 2022 Apr 15;22:329. doi: 10.1186/s12877-022-02989-6 (PMC9013054; doi:10.1186/s12877-022-02989-6)
Supplement: Supplementary file 3 — Additional file 3. [file 12877_2022_2989_MOESM3_ESM.docx]

**Additional File 3. Reasons for exclusion at full-text review stage**

**Legend**

| Objective not focused on postoperative transition in care (inclusive of care fragmentation) | 1 |
| --- | --- |
| Age | 2 |
| Non-elective | 3 |
| Commentary | 4 |
| Language | 5 |

| **Number** | **REF ID** | **Reference** | **Reason for Exclusion** |
| --- | --- | --- | --- |
| **Reports assessed for eligibility from electronic search** | | | |
| 1 | 20 | Justiniano, C. F., Xu, Z., Becerra, A. Z., Aquina, C. T., Boodry, C. I., Temple, L. K., & Fleming, F. J. (2019). Effect of care continuity on mortality of patients readmitted after colorectal surgery. *Journal of British Surgery*, *106*(5), 636-644. | 1 |
| 2 | 36 | Mousa, A. Y., Broce, M., Monnett, S., Davis, E., McKee, B., & Lucas, B. D. (2019). Results of telehealth electronic monitoring for post discharge complications and surgical site infections following arterial revascularization with groin incision. *Annals of vascular surgery*, *57*, 160-169. | 1 |
| 3 | 43 | Apostolakos, J. M., Boddapati, V., Fu, M. C., Erickson, B. J., Dines, D. M., Gulotta, L. V., & Dines, J. S. (2019). Continued Inpatient Care After Primary Total Shoulder Arthroplasty Is Associated With Increased Short-term Postdischarge Morbidity: A Propensity Score–Adjusted Analysis. *Orthopedics*, *42*(2), e225-e231. | 1 |
| 4 | 124 | Fernandes-Taylor, S., Berg, S., Gunter, R., Bennett, K., Smith, M. A., Rathouz, P. J., ... & Kent, K. C. (2018). Thirty-day readmission and mortality among Medicare beneficiaries discharged to skilled nursing facilities after vascular surgery. *Journal of Surgical Research*, *221*, 196-203. | 1 |
| 5 | 238 | Fu, M. C., Samuel, A. M., Sculco, P. K., MacLean, C. H., Padgett, D. E., & McLawhorn, A. S. (2017). Discharge to inpatient facilities after total hip arthroplasty is associated with increased postdischarge morbidity. *The Journal of arthroplasty*, *32*(9), S144-S149. | 1 |
| 6 | 278 | Graboyes, E. M., Kallogjeri, D., Saeed, M. J., Olsen, M. A., & Nussenbaum, B. (2017). Postoperative care fragmentation and thirty‐day unplanned readmissions after head and neck cancer surgery. *The Laryngoscope*, *127*(4), 868-874. | 1 |
| 7 | 380 | Schoenfeld, A. J., Zhang, X., Grabowski, D. C., Mor, V., Weissman, J. S., & Rahman, M. (2016). Hospital-skilled nursing facility referral linkage reduces readmission rates among Medicare patients receiving major surgery. *Surgery*, *159*(5), 1461-1468. | 1 |
| 8 | 441 | Szöts, K., Konradsen, H., Solgaard, S., Bogø, S., & Østergaard, B. (2015). Nurse‐led telephone follow‐up after total knee arthroplasty–content and the patients’ views. *Journal of clinical nursing*, *24*(19-20), 2890-2899. | 1 |
| 9 | 464 | Tousignant, M., Moffet, H., Nadeau, S., Mérette, C., Boissy, P., Corriveau, H., ... & Dimentberg, R. (2015). Cost analysis of in-home telerehabilitation for post-knee arthroplasty. *Journal of medical Internet research*, *17*(3), e3844. | 1 |
| 10 | 469 | Green, U. R., Dearmon, V., & Taggart, H. (2015). Improving transition of care for veterans after total joint replacement. *Orthopaedic Nursing*, *34*(2), 79-86. | 1 |
| 11 | 488 | Tsai, T. C., Orav, E. J., & Jha, A. K. (2015). Care fragmentation in the postdischarge period: surgical readmissions, distance of travel, and postoperative mortality. *JAMA surgery*, *150*(1), 59-64. | 2 |
| 12 | 528 | Sanford, D. E., Olsen, M. A., Bommarito, K. M., Shah, M., Fields, R. C., Hawkins, W. G., ... & Linehan, D. C. (2014). Association of discharge home with home health care and 30-day readmission after pancreatectomy. *Journal of the American College of Surgeons*, *219*(5), 875-886. | 1 |
| 13 | 778 | Holland, D. E., Mistiaen, P., & Bowles, K. H. (2011). Problems and unmet needs of patients discharged “home to self-care”. *Professional Case Management*, *16*(5), 240-250. | 2 |
| 14 | 839 | Harrison, J. D., Young, J. M., Auld, S., Masya, L., Solomon, M. J., & Butow, P. N. (2011). Quantifying postdischarge unmet supportive care needs of people with colorectal cancer: a clinical audit 1. *Colorectal Disease*, *13*(12), 1400-1406. | 1 |
| 15 | 869 | Soler, R. S., Juvinyà Canal, D., Noguer, C. B., Poch, C. G., Brugada Motge, N., & del Mar Garcia Gil, M. (2010). Continuity of care and monitoring pain after discharge: patient perspective. *Journal of advanced nursing*, *66*(1), 40-48. | 3 |
| 16 | 1265 | Bonevski, B., Doran, C., Bailey, C., & Lowe, J. (2002). Description of an early discharge post-acute care program: length of hospital stay, patient and carer needs and cost. *Australian Health Review*, *25*(2), 78-86. | 1 |
| 17 | 1480 | Gilliss, C. L., Gortner, S. R., Hauck, W. W., Shinn, J. A., Sparacino, P. A., & Tompkins, C. (1993). A randomized clinical trial of nursing care for recovery from cardiac surgery. *Heart & lung: the journal of critical care*, *22*(2), 125-133. | 1 |
| 18 | 1561 | Podolsky, R., & Mason, J. H. (1980). Geriatric discharge planning and follow-up. *IMJ. Illinois medical journal*, *157*(5), 291-292. | 1 |
| 19 | 1572 | Guilcher, S., Everall, A., Wodchis, W., deGraaf-Dunlop, J., Bar-Ziv, S., & Kuluski, K. (2019). Understanding Transitions of Care in Older Adults With Hip Fractures: A Multiple-Case Study in Ontario. *Archives of Physical Medicine and Rehabilitation*, *100*(10), e138. | 3 |
| 20 | 1579 | Rathbun, J. R., Ge, B., Mehr, D. R., Kruse, R. L., & Murray, K. S. (2019). Readmission after Radical Cystectomy Based on Discharge Destination. *Journal of the American College of Surgeons*, *229*(4), S322. | 1 |
| 21 | 1871 | Rojanasarot, S. (2018). The impact of early involvement in a postdischarge support program for ostomy surgery patients on preventable healthcare utilization. *Journal of Wound, Ostomy, and Continence Nursing*, *45*(1), 43. | 1 |
| 22 | 1606 | Wang, A., Li, Z., Rymer, J. A., Kosinski, A. S., Yerokun, B., Cox, M. L., ... & Vemulapalli, S. (2019). Relation of postdischarge care fragmentation and outcomes in transcatheter aortic valve implantation from the STS/ACC TVT Registry. *The American journal of cardiology*, *124*(6), 912-919. | 1 |
| 23 | 1615 | Theologis, A. A., Lau, D., Dalle-Ore, C., Tsu, A., Deviren, V., & Ames, C. P. (2021). Costs and utility of post-discharge acute inpatient rehabilitation following adult spinal deformity surgery. *Spine Deformity*, *9*(3), 817-822. | 1 |
| 24 | 1640 | Balentine, C. J., Leverson, G., Vanness, D. J., Knight, S., Turan, J., Brown, C. J., ... & Bhatia, S. (2018). Selecting post-acute care settings after abdominal surgery: Are we getting it right?. *The American Journal of Surgery*, *216*(2), 260-266. | 1 |
| 25 | 1670 | Kyte, K., Ekstedt, M., Rustoen, T., & Oksholm, T. (2019). Longing to get back on track: Patients’ experiences and supportive care needs after lung cancer surgery. *Journal of clinical nursing*, *28*(9-10), 1546-1554. | 1 |
| 26 | 1695 | Fleischman, A. N., Austin, M. S., Purtill, J. J., Parvizi, J., & Hozack, W. J. (2018). Patients living alone can be safely discharged directly home after total joint arthroplasty: a prospective cohort study. *JBJS*, *100*(2), 99-106. | 1 |
| 28 | 1698 | Lee, M. C., Tsauo, J. Y., Chen, M. C., Chen, H. M., Shih, S. L., Kao, M. J., ... & Woung, L. C. (2019, April). A high intensive post-acute home care program for patients with fragile fracture after surgery. In *INTERNATIONAL JOURNAL OF CLINICAL PRACTICE* (Vol. 73). 111 RIVER ST, HOBOKEN 07030-5774, NJ USA: WILEY. | 3 |
| 29 | 1712 | Shetty, K.,Ypsilantis, E.,Raza, I.,Smedley, F.,Ellul, J. (2018). Early discharge teams complements enhanced recovery from colorectal surgery. Journal Unknown. | 1 |
| 30 | 1726 | Weintraub, W. S., Elliott, D., Fanari, Z., Ostertag-Stretch, J., Muther, A., Lynahan, M., ... & Steinberg, T. H. (2018). The impact of care management information technology model on quality of care after Coronary Artery Bypass Surgery:“Bridging the Divides”. *Cardiovascular Revascularization Medicine*, *19*(1), 106-111. | 3 |
| 31 | 1747 | Owens, J. M., Callaghan, J. J., Duchman, K. R., Bedard, N. A., & Otero, J. E. (2018). Short-term morbidity and readmissions increase with skilled nursing facility discharge after total joint arthroplasty in a medicare-eligible and skilled nursing facility–eligible patient cohort. *The Journal of arthroplasty*, *33*(5), 1343-1347. | 1 |
| 32 | 1791 | Cook, C., Coronado, R. A., Bettger, J. P., & Graham, J. E. (2018). The association of discharge destination with 30-day rehospitalization rates among older adults receiving lumbar spinal fusion surgery. *Musculoskeletal Science and Practice*, *34*, 77-82. | 1 |
| 33 | 1793 | Wei, D., Liu, X., Chen, Y., Lin, Q., & Zhang, M. (2016, November). The research of applying WeChat to transitional care for the postoperative breast cancer patients. In *Cancer Nursing* (Vol. 39, pp. S38-S39). TWO COMMERCE SQ, 2001 MARKET ST, PHILADELPHIA, PA 19103 USA: LIPPINCOTT WILLIAMS & WILKINS. | 1 |
| 34 | 1809 | Sivasundaram, L., Heidari, K. S., Alluri, R. K., Heckmann, N., McKnight, B., Hill, J. R., & George, F. (2018). Discharge destination after shoulder arthroplasty: an independent risk factor for readmission and complications. *JAAOS-Journal of the American Academy of Orthopaedic Surgeons*, *26*(7), 251-259. | 1 |
| 35 | 1840 | Barad, S. J., Howell, S. M., & Tom, J. (2018). Is a shortened length of stay and increased rate of discharge to home associated with a low readmission rate and cost-effectiveness after primary total knee arthroplasty?. *Arthroplasty Today*, *4*(1), 107-112. | 1 |
| 36 | 1850 | Coskun, H., Senture, C., & Ustunsoz, A. (2016). The effectiveness of discharge training for patients after cardiac surgery. *Rehabilitation Nursing*. | 1 |
| 37 | 1862 | Krol, M. L., Allen, C., Setji, N., Graham, A. J., Jenkins, M., Shepherd, T., ... & White, H. (2018, April). Health Optimization Program for Elders (HOPE)-Improving Transitions from Hospital to Skilled Nursing Facility. In *JOURNAL OF THE AMERICAN GERIATRICS SOCIETY* (Vol. 66, pp. S121-S121). 111 RIVER ST, HOBOKEN 07030-5774, NJ USA: WILEY. | 3 |
| 38 | 1908 | Sabeh, K. G., Rosas, S., Buller, L. T., Roche, M. W., & Hernandez, V. H. (2017). The impact of discharge disposition on episode-of-care reimbursement after primary total hip arthroplasty. *The Journal of arthroplasty*, *32*(10), 2969-2973. | 1 |
| 39 | 1910 | Justiniano, C. F., Xu, Z., Becerra, A. Z., Aquina, C. T., Boodry, C. I., Swanger, A., ... & Fleming, F. J. (2017). Long-term deleterious impact of surgeon care fragmentation after colorectal surgery on survival: continuity of care continues to count. *Diseases of the Colon & Rectum*, *60*(11), 1147-1154. | 1 |
| 40 | 1931 | McLawhorn, A. S., Fu, M. C., Schairer, W. W., Sculco, P. K., MacLean, C. H., & Padgett, D. E. (2017). Continued inpatient care after primary total knee arthroplasty increases 30-day post-discharge complications: a propensity score-adjusted analysis. *The Journal of arthroplasty*, *32*(9), S113-S118. | 1 |
| 41 | 1958 | Lerner, J.,Chitnis, A.,Bhattacharyya, S.,Holy, C. E. (2017). Discharge destination after total joint arthroplasty and post-acute care costs. Journal Unknown. | 1 |
| 42 | 1961 | Ponnusamy, K. E., Naseer, Z., El Dafrawy, M. H., Okafor, L., Alexander, C., Sterling, R. S., ... & Skolasky, R. L. (2017). Post-discharge care duration, charges, and outcomes among Medicare patients after primary total hip and knee arthroplasty. *JBJS*, *99*(11), e55. | 1 |
| 43 | 1989 | Ypsilantis, E.,Archibald, H.,Smedley, F.,Ellul, J. (2017).  Post-operative nursing care at home: An invaluable adjunct to a successful enhanced recovery programme. Journal Uknown, | 1 |
| 44 | 1990 | Poch, L. C.,Fernandez, C. C.,Ruiz, M. G.,Bezanilla, S. C.,Tejon, G.,Pajaron, M.,Trabanco, S.,Martin, J. A.,Fleitas, M. G.,Diego, J. D. C. (2017). Home hospitalization care as support of enhanced recovery after surgery (ERAS). Our experience in 100 patients. Journal Unknown. | 1 |
| 45 | 2002 | Sween, R. R., & Grey, C. (2017, May). Interprofessional Geriatric Consultation to Improve Vulnerable Acute Care Patient Outcomes. In *JOURNAL OF THE AMERICAN GERIATRICS SOCIETY* (Vol. 65, pp. S207-S207). 111 RIVER ST, HOBOKEN 07030-5774, NJ USA: WILEY. | 1 |
| 46 | 2126 | Jorgenson, E. S., Richardson, D. M., Thomasson, A. M., Nelson, C. L., & Ibrahim, S. A. (2015). Race, rehabilitation, and 30-day readmission after elective total knee arthroplasty. *Geriatric orthopaedic surgery & rehabilitation*, *6*(4), 303-310. | 1 |
| 47 | 2169 | Todd, J., Ludwig, D., Simchuk, E., & Coates, A. (2015). Low hospital readmission rates achieved at rural bariatric center through education and a standardized discharge protocol. *Surgery for Obesity and Related Diseases*, *11*(6), S102-S103. | 1 |
| 48 | 2171 | Lauck, S. B., Wood, D. A., Baumbusch, J., Kwon, J. Y., Polderman, J., Cheung, A., ... & Webb, J. G. (2015). Standardized care to reduce length of stay and facilitate early discharge home after transcatheter aortic valve implantation: Implementation of the Vancouver clinical pathway. *Canadian Journal of Cardiology*, *31*(10), S115. | 1 |
| 49 | 2228 | Hall, M. H., Esposito, R. A., Pekmezaris, R., Lesser, M., Moravick, D., Jahn, L., ... & Hartman, A. R. (2014). Cardiac surgery nurse practitioner home visits prevent coronary artery bypass graft readmissions. *The Annals of thoracic surgery*, *97*(5), 1488-1495. | 3 |
| 50 | 2232 | Brooke, B. S., Stone, D. H., Cronenwett, J. L., Nolan, B., DeMartino, R. R., MacKenzie, T. A., ... & Goodney, P. P. (2014). Early primary care provider follow-up and readmission after high-risk surgery. *JAMA surgery*, *149*(8), 821-828. | 1 |
| 51 | 2274 | Schonberg, A., Rymarowicz, W., Cobelli, N., Cobelli, M., Ponnappan, S., & Joachim, P. (2014). Home Care: Discharge disposition for the older adult after elective joint replacement surgery: B187. *Journal of the American Geriatrics Society*, *62*. | 1 |
| 51 | 2329 | Şendir, M., Büyükyılmaz, F., & Muşovi, D. (2013). Patients’ Discharge information needs after total hip and knee arthroplasty: A quasi‐qualitative pilot study. *Rehabilitation nursing*, *38*(5), 264-271. | 1 |
| 52 | 2335 | Fredericks, S., & Yau, T. (2013). Educational intervention reduces complications and rehospitalizations after heart surgery. *Western journal of nursing research*, *35*(10), 1251-1265. | 1 |
| 53 | 2413 | Walke, L., Rosenthal, R., Perkal, M., Jeffery, S., Maiaroto, M., & Marottoli, R. (2012). D126: Identification of medication discrepancies in discharge paperwork among patients in the CO-OPERATE Geriatrics/Surgery comanagement program. *Journal of the American Geriatrics Society*, *60*. | 1 |
| 54 | 2552 | Ong, M., Davidson, B., Black, J., Mangione, C., Malik, S., Escarce, J., ... & Greenfield, S. (2010, June). READMISSION RANKS FOR HEART FAILURE PATIENTS: ARE THEY RELATED TO RANKS ON RESOURCE USE AND MORTALITY?. In *JOURNAL OF GENERAL INTERNAL MEDICINE* (Vol. 25, pp. 379-380). 233 SPRING ST, NEW YORK, NY 10013 USA: SPRINGER. | 1 |
| 55 | 2559 | Legner, V. J., Massarweh, N. N., Symons, R. G., McCormick, W. C., & Flum, D. R. (2009). The significance of discharge to skilled care after abdominopelvic surgery in older adults. *Annals of surgery*, *249*(2), 250-255. | 1 |
| 56 | 2583 | Harrison, J., Young, J., Auld, S., Solomon, M., Butow, P., & Masya, L. (2009). QUANTIFYING PATIENTS'SUPPORTIVE CARE NEEDS: A CLINICAL AUDIT OF NON-ADMITTED PATIENT OCCASIONS OF SERVICE: 191. *Asia-pacific Journal of Clinical Oncology*, *5*. | 1 |
| 57 | 2599 | Cebeci, F., & Çelik, S. Ş. (2008). Discharge training and counselling increase self‐care ability and reduce postdischarge problems in CABG patients. *Journal of clinical nursing*, *17*(3), 412-420. | 1 |
| 58 | 2612 | Mcmurray, A., Johnson, P., Wallis, M., Patterson, E., & Griffiths, S. (2007). General surgical patients’ perspectives of the adequacy and appropriateness of discharge planning to facilitate health decision‐making at home. *Journal of clinical nursing*, *16*(9), 1602-1609. | 1 |
| 59 | 2615 | Kleinpell, R. M., & Avitall, B. (2007). Integrating telehealth as a strategy for patient management after discharge for cardiac surgery: results of a pilot study. *Journal of Cardiovascular Nursing*, *22*(1), 38-42. | 1 |
| 60 | 2749 | Heine, J., Koch, S., & Goldie, P. (2004). Patients' experiences of readiness for discharge following a total hip replacement. *Australian Journal of Physiotherapy*, *50*(4), 227-233. | 1 |
| 61 | 2800 | Lam, P., White, C. L., Runions, S., & Miller, C. A. (2001). Continuity of care for short-stay neurosurgery patients: a quality improvement initiative. *Axone (Dartmouth, NS)*, *23*(2), 14-21. | 2 |
| 62 | 2808 | Rutherford, A., & Burge, B. (2001). General practitioners and hospitals. Continuity of care. *Australian family physician*, *30*(11), 1101-1107. | 2 |
| 63 | 2855 | Kelly, M. H., & Ackerman, R. M. (1999). Total joint arthroplasty: a comparison of postacute settings on patients functional outcomes. *Orthopaedic Nursing*, *18*(5), 75. | 1 |
| 64 | 2889 | Bert, J., & Killeen, K. (1998). A comparison of hospital length of stay versus the early transfer to a skilled nursing facility: the effect on direct costs of total joint arthroplasty. *The Knee*, *5*(1), 49-52. | 1 |
| 65 | 2904 | Von Sternberg, T., Hepburn, K., Cibuzar, P., Convery, L., Dokken, B., Haefemeyer, J., ... & Won‐Savage, R. (1997). Models of Geriatrics Practice: Post‐Hospital Sub‐Acute Care: An Example of a Managed Care Model. *Journal of the American Geriatrics Society*, *45*(1), 87-91. | 3 |
| 66 | 2921 | Galloway, S. C., & Graydon, J. E. (1996). Uncertainty, symptom distress, and information needs after surgery for cancer of the colon. *Cancer nursing*, *19*(2), 112-117. | 1 |
| 67 | 3000 | Ford, M.,Wasilewicz, C. (1981). Bridging the gap between hospital and home. Journal Unknown. | 4 |
| 68 | 3107 | Forouzesh, M., Sanagoo, A., Vakili, M. A., & Jouybari, L. (2017). The effect of telenursing (telephone follow up) after discharge on readmission due to complications after coronary artery bypass graft surgery. *Nursing And Midwifery Journal*, *15*(8), 584-594. | 5 |
| 69 | 3411 | Causey-Upton, R., & Howell, D. M. (2017). Patient experiences when preparing for discharge home after total knee replacement. *Internet Journal of Allied Health Sciences and Practice*, *15*(1), 5. | 1 |
| 70 | 3430 | Llagostera Sillano Gentil, Luana,Siqueira Costa, Ana Lucia.  (2016). Discharge Planning After Myocardial Revascularization. Journal Unknown. | 1 |
| 71 | 3477 | Jubelt, L. E., Goldfeld, K. S., Chung, W. Y., Blecker, S. B., & Horwitz, L. I. (2016). Changes in discharge location and readmission rates under Medicare bundled payment. *JAMA internal medicine*, *176*(1), 115-117. | 1 |
| 72 | 3494 | Reay, P. A., Horner, B., & Duggan, R. (2015). The patient's experience of early discharge following total hip replacement. *International journal of orthopaedic and trauma nursing*, *19*(3), 131-139. | 1 |
| 73 | 3506 | Lithner, M., Klefsgard, R., Johansson, J., & Andersson, E. (2015). The significance of information after discharge for colorectal cancer surgery–a qualitative study. *BMC nursing*, *14*(1), 1-8. | 1 |
| 74 | 3542 | Healthy Transitions from Hospital to Skilled Nursing Facility Post-CABG | 3 |
| 75 | 3673 | 'If I didn't have anybody, what would I have done?': Experiences of older adults and their discharge home after lower limb orthopaedic surgery | 1 |
| 76 | 3690 | Lapum, J., Angus, J. E., Peter, E., & Watt-Watson, J. (2011). Patients' discharge experiences: returning home after open-heart surgery. *heart & lung*, *40*(3), 226-235. | 1 |
| 77 | 3703 | Hørdam, B., Pedersen, P. U., Søballe, K., Sabroe, S., & Ehlers, L. H. (2011). Quality-adjusted life years gained in patients aged over 65 years after total hip replacement. *International Journal of Orthopaedic and Trauma Nursing*, *15*(1), 11-17. | 1 |
| 78 | 3707 | Spyropoulos, V., Ampleman, S., Miousse, C., & Purden, M. (2011). Cardiac surgery discharge questionnaires: meeting information needs of patients and families. *Canadian Journal of Cardiovascular Nursing*, *21*(1). | 1 |
| 79 | 3747 | Tanner, J., Khan, D., Aplin, C., Ball, J., Thomas, M., & Bankart, J. (2009). Post-discharge surveillance to identify colorectal surgical site infection rates and related costs. *Journal of Hospital Infection*, *72*(3), 243-250. | 1 |
| 80 | 3753 | Home continuity of care programme after discharge from a domiciliary hospitalisation unit | 5 |
| 81 | 3836 | Sheard, C., & Garrud, P. (2006). Evaluation of generic patient information: effects on health outcomes, knowledge and satisfaction. *Patient education and counseling*, *61*(1), 43-47. | 1 |
| 82 | 3844 | Supporting seniors after discharge...'Senior surgery' (Nov. 24, 2005) | 4 |
| 83 | 3877 | Theobald, K., & McMurray, A. (2004). Coronary artery bypass graft surgery: discharge planning for successful recovery. *Journal of advanced nursing*, *47*(5), 483-491. | 1 |
| 84 | 3908 | Paul, R. P., & Robichaud-Ekstrand, S. (2002). Expected and received assistance from informal social support for aged people in heart surgery. *Recherche en soins infirmiers*, (71), 38-55. | 1 |
| 85 | 3920 | Hölttä, R., Hupli, M., & Salanterä, S. (2002). Patients’ learning needs after coronary artery bypass surgery. *Hoitotiede*, *14*, 11-18. | 1 |
| 86 | 3935 | McCorkle, R., Nuamah, I. F., Strumpf, N. E., Adler, D. C., Cooley, M. E., Jepson, C., ... & Torosian, M. (2000). A specialized home care intervention improves survival among older post-surgical cancer patients. *JOURNAL-AMERICAN GERIATRICS SOCIETY*, *48*(12), 1707-1713. | 1 |
| 87 | 3967 | McNamee, S., & Wallis, M. (1999). Patient problems following discharge and evaluation of patient discharge education on a cardiac surgical ward. *Nursing Monograph*, *1999*. | 1 |
| 88 | 3973 | Boter, H., Mistiaen, P., Duijnhouwer, E., & Groenewegen, I. (1998). The problems of elderly patients at home after ophthalmic treatment. *Journal of ophthalmic nursing & technology*, *17*(2), 59-65. | 1 |
| 89 | 4036 | Beckie, T. (1989). A supportive-educative telephone program: impact on knowledge and anxiety after coronary artery bypass graft surgery. *Heart & lung: the journal of critical care*, *18*(1), 46-55. | 1 |
| 90 | 71 | Brauer, D., Wu, N., Keller, M., Humble, S., Hammill, C., Fields, R., ... & Sanford, D. (2020, February). Care Fragmentation in Readmissions Following Hepatopancreatobiliary and Gastric Oncologic Surgery is Associated with Increased Mortality: Identifying Patients At-Risk. In *ANNALS OF SURGICAL ONCOLOGY* (Vol. 27, No. SUPPL 1, pp. S41-S41). ONE NEW YORK PLAZA, SUITE 4600, NEW YORK, NY, UNITED STATES: SPRINGER. | 1 |
| 91 | 140 | Hirji, S. A., Zogg, C. K., Vaduganathan, M., Kiehm, S., Percy, E. D., Yazdchi, F., ... & Kaneko, T. (2020). Quantifying the Impact of Care Fragmentation on Outcomes After Transcatheter Aortic Valve Implantation. *The American Journal of Cardiology*, *128*, 113-119. | 1 |
| 92 | 191 | McGillion, M., Ouellette, C., Good, A., Bird, M., Henry, S., Clyne, W., ... & Devereaux, P. J. (2020). Postoperative Remote Automated Monitoring and Virtual Hospital-to-Home Care System Following Cardiac and Major Vascular Surgery: User Testing Study. *Journal of medical Internet research*, *22*(3), e15548. | 1 |
| 93 | 215 | Peralta, J., Ham, S. W., Magee, G. A., Lane, C., Johnson, C., Issai, A., ... & Weaver, F. A. (2020). Impact of a care delivery redesign initiative for vascular surgery. *Journal of vascular surgery*, *71*(2), 599-608. | 1 |
| 94 | 262 | Takchi, R., Williams, G. A., Brauer, D., Stoentcheva, T., Wolf, C., Van Anne, B., ... & Hawkins, W. G. (2020). Extending enhanced recovery after surgery protocols to the post-discharge setting: a phone call intervention to support patients after expedited discharge after pancreaticoduodenectomy. *The American Surgeon*, *86*(1), 42-48. | 1 |
| 95 | 282 | Xu, Y., Li, S., Zhao, P., & Zhao, J. (2020). Using the knowledge-to-action framework with joint arthroplasty patients to improve the quality of care transition: a quasi-experimental study. *Journal of orthopaedic surgery and research*, *15*(1), 1-5. | 2 |
| 96 | 303 | Ko, Y., Lee, J., Oh, E., Choi, M., Kim, C., Sung, K., & Baek, S. (2019). Older adults with hip arthroplasty: an individualized transitional care program. *Rehabilitation Nursing Journal*, *44*(4), 203-212. | 3 |
| 97 | 307 | Beal, E. W., Bagante, F., Paredes, A., Chen, Q., Akgul, O., Merath, K., ... & Pawlik, T. M. (2019). Index versus non-index readmission after hepato-pancreato-biliary surgery: where do patients go to be readmitted?. *Journal of Gastrointestinal Surgery*, *23*(4), 702-711. | 1 |
| 98 | 320 | Patient-centered quality of transitional care for hospitalized patients and its influencing factors | 3 |
| 99 | 362 | Kothari, A. N., Yau, R. M., Blackwell, R. H., Schaidle-Blackburn, C., Markossian, T., Zapf, M. A., ... & Kuo, P. C. (2016). Inpatient rehabilitation after liver transplantation decreases risk and severity of 30-day readmissions. *Journal of the American College of Surgeons*, *223*(1), 164-171. | 3 |
| 100 | 421 | Wong, Z. W., Maciver, S., Khushu, A., Rabbidge, L., Bucki, T., Ganju, V., ... & Velandai, S. (2019, November). Survivorship needs of an elderly population with cancer: A pilot study of care coordination. In *ASIA-PACIFIC JOURNAL OF CLINICAL ONCOLOGY* (Vol. 15, pp. 154-154). 111 RIVER ST, HOBOKEN 07030-5774, NJ USA: WILEY. | 1 |
| 101 | 426 | Jolly Graham, A., et al. (2018). "Health optimization program for elders (HOPE)-improving transitions from hospital to skilled nursing facility. | 3 |
| 102 | 435 | Yang, H., Dervin, G., Madden, S., Beaulé, P. E., Gagné, S., Crossan, M. L., ... & Taljaard, M. (2018). Postoperative home monitoring after joint replacement: feasibility study. *JMIR Perioperative Medicine*, *1*(2), e10168. | 2 |
| 103 |  | Sinvani, L. D., Beizer, J., Akerman, M., Pekmezaris, R., Nouryan, C., Lutsky, L., ... & Wolf-Klein, G. (2013). Medication reconciliation in continuum of care transitions: a moving target. *Journal of the American Medical Directors Association*, *14*(9), 668-672. | 3 |
| **Reference Lists** | | | |
| 104 | Ref list 1 | Naylor, M. D., Brooten, D. A., Campbell, R. L., Maislin, G., McCauley, K. M., & Schwartz, J. S. (2004). Transitional care of older adults hospitalized with heart failure: a randomized, controlled trial. *Journal of the American Geriatrics Society*, *52*(5), 675-684. | 3 |
| 105 | Ref list 2 | Naylor, M. D., & McCauley, K. M. (1999). The effects of a discharge planning and home follow-up intervention on elders hospitalized with common medical and surgical cardiac conditions. *Journal of Cardiovascular Nursing*, *14*(1), 44-54. | 3 |
| 106 | Ref list 3 | Shyu, Y. I. L. (2000). The needs of family caregivers of frail elders during the transition from hospital to home: a Taiwanese sample. *Journal of advanced nursing*, *32*(3), 619-625. | 3 |
| 107 | Ref list 4 | Boockvar, K. S., Fridman, B., & Marturano, C. (2005). Ineffective communication of mental status information during care transfer of older adults. *Journal of General Internal Medicine*, *20*(12), 1146-1150. | 3 |
| 108 | Ref list 5 | Halasyamani, L., Kripalani, S., Coleman, E., Schnipper, J., Van Walraven, C., Nagamine, J., ... & Manning, D. (2006). Transition of care for hospitalized elderly patients—development of a discharge checklist for hospitalists. *Journal of Hospital Medicine*, *1*(6), 354-360. | 3 |
| 109 | Ref list 6 | Coleman, E. A., & Boult, C. (2003). Improving the quality of transitional care for persons with complex care needs. *Journal of the American Geriatrics Society*, *51*(4), 556-557. | 3 |
| 110 | Ref list 7 | Naylor, M. D., Brooten, D., Campbell, R., Jacobsen, B. S., Mezey, M. D., Pauly, M. V., & Schwartz, J. S. (1999). Comprehensive discharge planning and home follow-up of hospitalized elders: a randomized clinical trial. *Jama*, *281*(7), 613-620. | 3 |
| 111 | Ref list 8 | Naylor, M., Brooten, D., Jones, R., Lavizzo-Mourey, R., Mezey, M., & Pauly, M. (1994). Comprehensive discharge planning for the hospitalized elderly: a randomized clinical trial. *Annals of internal Medicine*, *120*(12), 999-1006. | 1 |
| 112 | Ref list 9 | Cheek, J., Ballantyne, A., Gillham, D., Mussared, J., Flett, P., Lewin, G., ... & Vandermeulen, S. (2006). Improving care transitions of older people: challenges for today and tomorrow. *Quality in Ageing and Older Adults*. | 3 |
| **Clinical Trial Registries** | | | |
| 113 | 1 | NCT04236583: Improving the Hospital-to-Home Transition Through Post-Discharge Virtual Visits in Primary Care | 3 |
| 114 | 2 | NCT03181906: Effectiveness of Pre-Consultation Medication Reconciliation Service in Reducing Unintentional Medication Discrepancies During Transition of Care From Hospital Discharge to Primary Care Setting- A Randomised Controlled Trial | 3 |
| 115 | 3 | JPRN-UMIN000026528: Intervention study on improvement of care transition process using standardized care information sheet in home-bound frail elderly admission - Care transition process study using standardized care information sheet | 3 |
| 116 | 4 | ACTRN12605000376640: A randomised controlled trial to evaluate the effectiveness of a medication management service on medication appropriateness in people transferring to residential care for the first time | 1 |
| 117 | 5 | ISRCTN77474147: Bridging the transition from hospital to home: Effects of the VITAL Telehealth Program on recovery for CABG patients and their caregivers | 2 |
| 118 | 6 | RBR-44vznyc: Development, evaluation, and implementation of a Care Transition Strategy for patients with Colorectal Cancer | 2 |
| 119 | 7 | NCT03832257: Improving Safety of Transitions to Skilled Nursing Care Using Videoconferencing (ECHO-CT) | 3 |
| 120 | 9 | NCT04136951: Improving Patient Prioritization During Hospital-homecare Transition (PREVENT) | 3 |
