# Supplementary material for: Evaluations of postoperative transitions in care for older adults: a scoping review
Source: BMC Geriatr. 2022 Apr 15;22:329. doi: 10.1186/s12877-022-02989-6 (PMC9013054; doi:10.1186/s12877-022-02989-6)
Supplement: Supplementary file 4 — Additional file 4. [file 12877_2022_2989_MOESM4_ESM.docx]

| **Additional File 4. Intervention Descriptions** | |
| --- | --- |
| **Author/Study Design** | **Intervention Description** |
| Missel et al., 2015;  Quasi-experimental intervention study | - Rehabilitation counselling the day before discharge that included: how to manage and cope with physical, psychological, social and existential/ spiritual/religious problems and challenges during and after cancer treatment - Counselling included lifestyle changes such as smoking cessation, increased physical activity, limited alcohol intake and healthy diet - Counselling based on a booklet that patients received the day before, starting with areas most important to the patients - Patients were informed about where to seek help after discharge |
| Sawatzky et al., 2013; Randomized clinical trial, two group, repeated measures design | - A Nurse Practitioner conducted a needs assessment via telephone follow-up approximately 3 days post-discharge   Patients with significant issues/concerns were seen in the Nurse Practitioner Follow-Up (NPFU) clinic |
| Young et al., 2013; Two-arm, parallel-group multi-center randomized trial | - CONNECT is supplementary to usual follow-up care and involves no face-to-face contact - It consists of 5 scheduled, structured telephone calls on days 3 and 10 and then at 1, 3, and 6 months after hospital discharge - CONNECT aims to improve patients’ ability to navigate the health system for clinical and supportive care, and to provide information and emotional support directly |
| Middleton et al., 2004; Randomized controlled trial | - Patients received telephone contact by a Registered Nurse at 2, 6 and 12 weeks at which time stroke risk factors were reviewed - Between telephone contacts, patients were mailed information about stroke risk factors and management - Nurse phoned each General Practitioner to inform them of their patient’s immediate postoperative recovery - Surgeon and General Practitioner were faxed individual summaries for each patient outlining stroke risk factors s/he was interested in changing |
| McDonald et al., 2018; Cohort study | - The Perioperative Optimization of Senior Health (POSH) intervention is a coordinated interdisciplinary perioperative care program - During the preoperative visit, the POSH team offered recommendations for risk-reducing strategies in the pre- and post-operative periods and anticipating needs at discharge; the hospital Geriatrics Consult Team followed patients daily - Physicians who conducted preoperative POSH evaluations also participated in rounds on the inpatient Geriatrics Consult Service - The geriatrics and surgery teams jointly counselled patients and families, helping them prepare for discharge and posthospital care |
| Shargall et al., 2016; Pilot retrospective cohort study | - Hospital-operated Integrated Comprehensive Care Program - Interdisciplinary team - Nurse coordinator assesses patients, conducts discharge planning (based on care pathways and family input) and supervises the coordination of the outpatient home care team - All patient data electronically sent to home care team who contacted patients within 24 hours of being home - Patient recovery details uploaded to mobile devices by home care team to facilitate continuity of care |
| Quinlan et al., 2020; Quality Improvement project | - An adaptable smart phone or tablet design technology offered to patients in follow-up in addition to interactive voice response, email and texting options, based on patient choice |
| Wong et al., 2018; Pilot qualitative study | - Targeted discharge education (self-care education, discharge tools, confirmation of discharge location and contact information) delivered by an Advanced Practice Nurse - Series of follow-up telephone calls with structured interviews focused on key areas of transitional care risk including: review of medications, identification of symptoms and emphasis of early follow-up care |
| Hughes et al., 2000; Descriptive qualitative study | - Home care intervention including 3 home visits and 5 telephone contacts from an Advanced Practice Nurse during the first 4 weeks after hospital discharge |
