# Supplementary material for: Evaluations of postoperative transitions in care for older adults: a scoping review
Source: BMC Geriatr. 2022 Apr 15;22:329. doi: 10.1186/s12877-022-02989-6 (PMC9013054; doi:10.1186/s12877-022-02989-6)
Supplement: Supplementary file 5 — Additional file 5. [file 12877_2022_2989_MOESM5_ESM.docx]

| **Additional File 5. Process Evaluation Descriptions** | |
| --- | --- |
| **Author/Study Design** | **Process Evaluation Description** |
| Missel et al 2015; Quasi-experimental intervention study | The following aspects of intervention implementation were evaluated using a questionnaire: Patient knowledge of rehabilitation during the transition, number of patients offered physical rehabilitation referrals, counselling to discuss financial or work-related problems, offer of psychological assistance, invitation to speak with other lung cancer patients, support regarding religious considerations, knowledge of how to seek support after discharge, knowledge of where to address questions about the disease or treatment after discharge, knowledge of other places than the hospital to seek help, feeling of safety at discharge, knowledge about importance of lifestyle for health, whether the nurses took care of individual needs at discharge. |
| Middleton et al 2004; Randomized Controlled Trial | The following aspects of intervention implementation were evaluated using a questionnaire: Patient knowledge of stroke risk factors, patient recollection of information received about stroke and stroke risk factor management since their operation. |
| Shargall et al 2016; Pilot retrospective cohort study | The following aspects of intervention implementation were evaluated using a questionnaire: Program uptake including homecare services used; percentage who used the telephone support provided by the program coordinator. |
| Xourafas et al 2016; Observational cohort study | Medical records and surveys were used to identify predictive factors including but not limited to: communication, social support, understanding discharge instructions, compliance with discharge instructions, and inpatient and outpatient management were evaluated as variables to predict readmission. |
| Weinberg et al 2007; Prospective cohort study | Caregiver-reported interactions with formal providers. Caregiver preparation to provide care after surgery. Evaluated using surveys. |
| Weinberg et al 2007; Prospective cohort study | Provider-reported challenges with coordination; patient-reported coordination of care problems. |
| Brooke et al 2019; Qualitative interview study | Concordance of information between patients, surgical providers and primary care providers during transitions of surgical care. Evaluated using interviews. |
| Wong et al 2018; Pilot qualitative study | The following aspects of intervention implementation were evaluated using a questionnaire: Medication reconciliation; patient-reported difficulty/ease of accessing their family doctor; adherence to tracking weight; ability to do usual activities; new or worsened symptoms; percentage of patients who were reached for the telephone calls to support transitional care. |
| Slager et al 2017; Qualitative interview study | Strategies to optimize provider-provider communication using interviews. |
| Hughes et al 2000; Descriptive qualitative study | Patient information needs during the transition from hospital to home based on nursing documentation of what interventions they provided during home-care. |
